# Covalent Organic Framework (COF) Derived Ni‐N‐C Catalysts for Electrochemical CO_2_ Reduction: Unraveling Fundamental Kinetic and Structural Parameters of the Active Sites

**DOI:** 10.1002/anie.202114707

**Published:** 2022-02-16

**Authors:** Changxia Li, Wen Ju, Sudarshan Vijay, Janis Timoshenko, Kaiwen Mou, David A. Cullen, Jin Yang, Xingli Wang, Pradip Pachfule, Sven Brückner, Hyo Sang Jeon, Felix T. Haase, Sze‐Chun Tsang, Clara Rettenmaier, Karen Chan, Beatriz Roldan Cuenya, Arne Thomas, Peter Strasser

**Affiliations:** ^1^ Department of Chemistry Division of Functional Materials Technical University Berlin Berlin 10623 Germany; ^2^ Department of Chemistry Chemical Engineering Division Technical University Berlin Berlin 10623 Germany; ^3^ CatTheory Department of Physics Technical University of Denmark 2800 Kongens Lyngby Denmark; ^4^ Interface Science Department Fritz-Haber Institute of Max-Planck Society Berlin 14195 Germany; ^5^ Center for Nanophase Materials Sciences Oak Ridge National Laboratory Oak Ridge TN USA

**Keywords:** Active Site Density, CO2 Reduction, Covalent Organic Framework, Single-Site Ni-N-C, Turnover Frequency

## Abstract

Electrochemical CO_2_ reduction is a potential approach to convert CO_2_ into valuable chemicals using electricity as feedstock. Abundant and affordable catalyst materials are needed to upscale this process in a sustainable manner. Nickel‐nitrogen‐doped carbon (Ni‐N‐C) is an efficient catalyst for CO_2_ reduction to CO, and the single‐site Ni−N_*x*_ motif is believed to be the active site. However, critical metrics for its catalytic activity, such as active site density and intrinsic turnover frequency, so far lack systematic discussion. In this work, we prepared a set of covalent organic framework (COF)‐derived Ni‐N‐C catalysts, for which the Ni−N_*x*_ content could be adjusted by the pyrolysis temperature. The combination of high‐angle annular dark‐field scanning transmission electron microscopy and extended X‐ray absorption fine structure evidenced the presence of Ni single‐sites, and quantitative X‐ray photoemission addressed the relation between active site density and turnover frequency.

## Graphical abstract

Nickel–nitrogen‐doped carbon (Ni‐N‐C) is an efficient catalyst for electrochemical CO_2_ reduction to CO, yet methods to evaluate the number of active Ni sites on the surface and turnover frequency remained elusive. Herein, a set of COF‐derived Ni‐N‐C candidates is synthesized and a comprehensive analysis is undertaken to quantify the active Ni sites, addressing the relation between synthesis parameters, apparent reactivity, active site density, and turnover frequency.

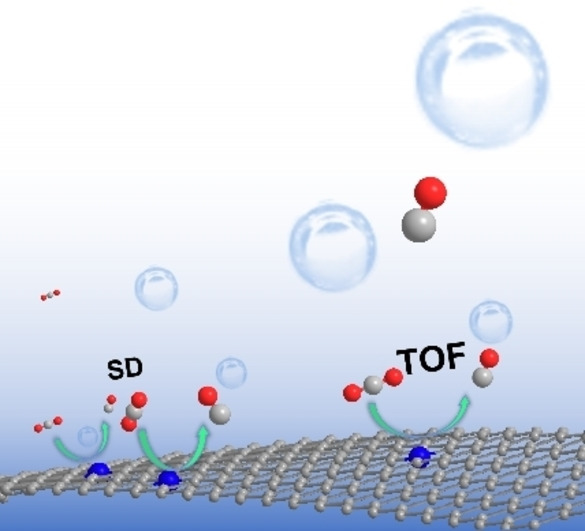

## Introduction

The direct electrochemical CO_2_ reduction reaction (CO_2_RR) to value‐added chemicals and fuels is a potential pathway to reduce CO_2_ emissions.^[1]^ However, the CO_2_RR is commonly accompanied by water splitting; hence, the hydrogen evolution reaction (HER) is strongly competing.^[2]^ Moreover, the CO_2_RR process yields a wide range of carbon‐based products as CO, formate, hydrocarbons, and oxygenates,^[3]^ which require additional product separation in the practical implementation. Therefore, an affordable and selective catalyst is urgently needed to drive this electrochemical conversion efficiently.

In previous investigations, a great variety of catalysts have been studied for CO_2_RR.[^2a^, ^4^] Among them, the class of non‐precious metal‐nitrogen doped carbon (M‐N‐C) catalysts has received particular attention.^[5]^ These catalysts feature graphene‐embedded, single‐site M−N_*x*_ structural motifs, which allow efficient CO_2_ to CO conversion.^[6]^ Unlike extended metal surfaces, the single‐site M−N_*x*_ moieties in the M‐N‐C catalysts enable the suppression of the HER side reaction, yielding highly selective catalysis and high purity CO streams.[^2b^, ^6c^, ^7^] The Ni‐functionalized versions (Ni‐N‐Cs) attracted attention due to their exceptional performance and selectivity to CO. Over 90 % CO selectivity could be achieved on these Ni‐N‐C catalysts, even at industry‐relevant currents.[^6a^, ^6b^, ^6c^, ^6d^, ^6e^, ^6g^, ^8^] Our current understanding of this catalytic reaction attributes this unique reactivity to the chemical nature of the embedded Ni−N_*x*_ motifs. A key hypothesis to explain the reactivity states that under CO_2_RR conditions, the Ni−N_*x*_ site binds weakly to *H (diminished proton uptake competition) and *CO (minor CO poisoning issue), enabling the CO_2_‐to‐CO cascade at reduced kinetic barriers. Therefore, a reasonable turnover frequency (TOF) could be realized at sufficiently applied cathode potentials.[^6a^, ^6g^] However, this hypothesis about the origin of the high catalytic reactivity still awaits experimental verification at the molecular level, nor has the Ni‐N‐C CO_2_ to CO catalytic reactivity ever been fundamentally deconvoluted into the two basic kinetic descriptors of surface active site density (SD) and intrinsic TOF. This contribution will address these points.

In M‐N‐C single metal atom site electrocatalysts, carbon is the main constituent element. Its porous structure provides a large electrochemical surface area and tunable chemical composition, benefiting the loading of active M−N_*x*_ moieties. To date, a number of distinct synthesis strategies have been established to generate M‐N‐C catalysts. These strategies involve catalyst precursors as diverse as supported macrocyclic compounds,[^6e^, ^9^] polymers,^[7a]^ zeolitic imidazolate frameworks (ZIF),^[10]^ or covalent and metal organic frameworks (COFs and MOFs, respectively).[^6d^, ^11^]

Generally, the catalyst's apparent catalytic mass activity (MA) is contingent on two basic descriptors: the intrinsic catalytic TOF and the active SD. The MA is linked according to MA=TOF×SD.[^6f^, ^12^] To effectively improve the CO_2_RR performance of M‐N‐C catalysts, one approach is to select the active site and a suitable potential window for an optimal TOF, while the other is to increase the effective SD. In previous studies on pyrolyzed M‐N‐C CO_2_RR catalysts, only the active sites’ nature (i.e., TOF‐related) has been addressed, whereas the SD descriptor still lacks systematic exploration and discussion.

Herein, we study and unravel the molecular structure, composition, and CO_2_ reduction reaction mechanism of new COF‐derived Ni‐N‐Cs. The experimental MA of the Ni‐N‐C catalysts has been deconvoluted into SD and TOF of the surface‐active Ni−N_*x*_ sites. Finally, these fundamental experimental descriptors have been correlated to corresponding computational results derived from first principle density functional theory (DFT). Through the first principle calculations and comparisons of intrinsic TOF and Tafel slopes, we were able to attribute the mean N coordination of the Ni−N_*x*_ motifs and conclude on the chemical structure of the active sites.

The COF precursor material in focus is a nitrogen‐rich triazole‐based COF (named TpDt‐COF and TpDt‐COF‐Ni after Ni loading). In general, COFs are ideal materials to anchor coordinative metal sites precisely due to their ordered porous structure and tunable composition.^[13]^ The structural features can be partially preserved when the metal‐coordinated COF is heated to higher temperatures and carbonized.^[13d]^ The COF‐derived Ni‐N‐C catalysts are referred to as C‐TpDt‐Ni. After pyrolysis and acid washing, the catalyst C‐TpDt‐Ni‐900 (treated at 900 °C) shows above 90 % faradaic CO efficiency at approximately 25 mA cm^−2^ current density, measured in the regular liquid H‐cell. High angle annular dark‐field scanning transmission electron microscopy (HAADF‐STEM) and extended X‐ray absorption fine structure (EXAFS) were performed to evidence the single‐atom nature of the Ni active sites. X‐ray photoemission (XPS) analysis was carried out and yielded quantitative catalyst SD for the Ni‐N‐C catalysts. Linking the quantitative experimental fittings to DFT calculations and micro‐kinetic modelling, a mechanistic relationship between the synthesis condition, SD, TOF, and their apparent MA for CO_2_RR could be provided.

## Synthesis and Characterization

### Synthesis of the TpDt‐COF‐Ni Precursor

**Figure 1:**
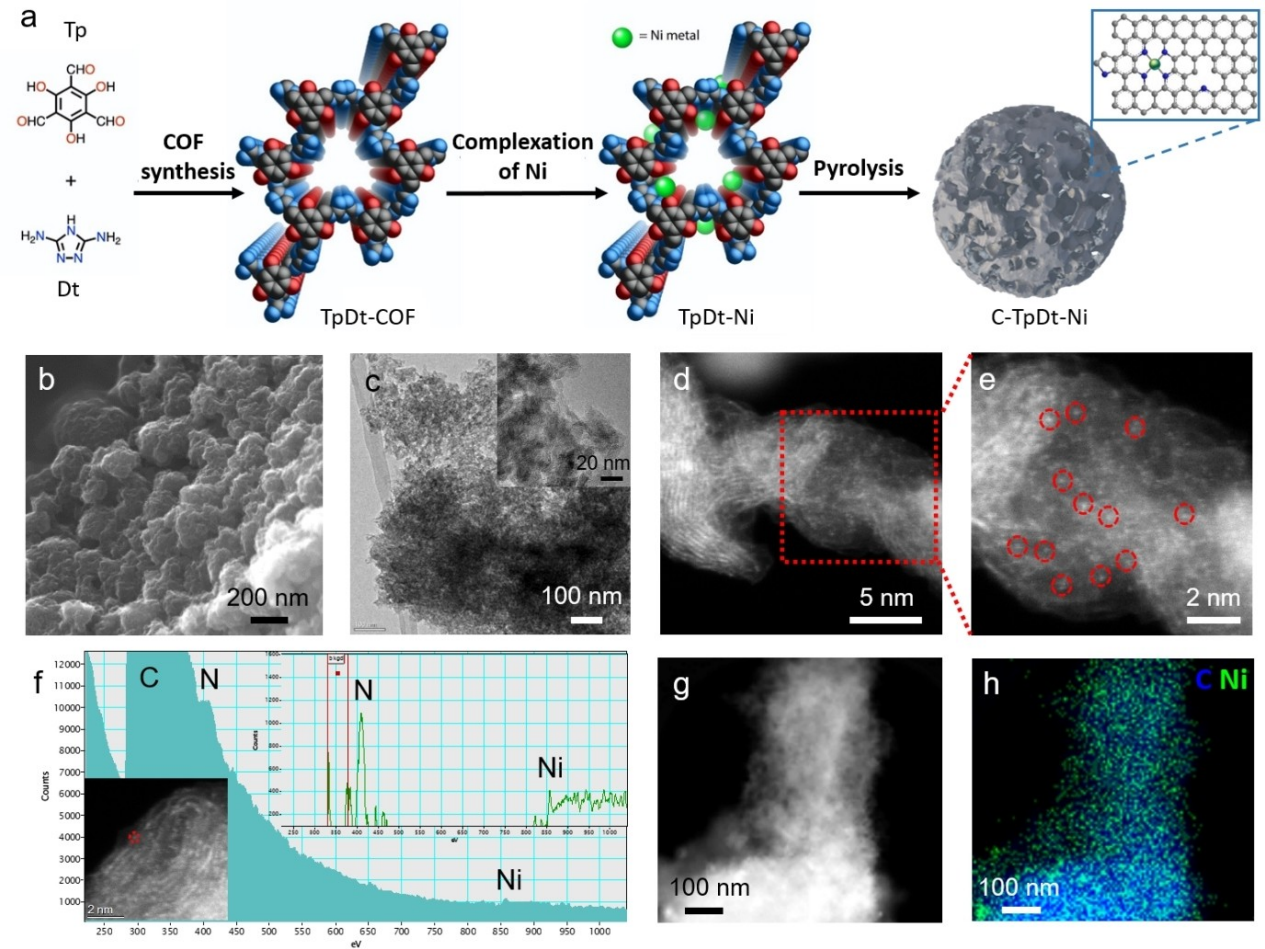
a) Scheme for the synthesis of C‐TpDt‐Ni (carbon atoms are shown in grey, nitrogen in blue, oxygen in red, Ni in green; hydrogen atoms are not shown). b) SEM image and c) TEM image of C‐TpDt‐Ni‐900. d, e) High‐resolution HAADF‐STEM image of C‐TpDt‐Ni‐900. f) EELS spectrum acquired at Ni single‐atom region circled in red. g, h) HAADF‐STEM image and corresponding EDS mapping image of C‐TpDt‐Ni‐900.

A triazole‐based COF (TpDt‐COF) was selected as the catalyst precursor because of its high nitrogen content.^[14]^ The detailed synthesis route is given in the Supporting Information. In brief, the TpDt‐framework was prepared by the condensation polymerization of 1,3,5‐tri‐formyl‐phloroglucinol (Tp) and 3,5‐diamino‐1,2,4‐triazole (Dt) via a solvothermal method (Figure 1a and Figure S1), delivering a nitrogen‐rich COF backbone with high porosity. Ni impregnation was performed by dispersing the TpDt‐COF in a Ni(NO_3_)_2_⋅6 H_2_O aqueous solution. The Ni ions can coordinate to the heteroatoms of the backbone and are thus uniformly dispersed in the COF. After removing excess Ni ions with distilled water, the TpDt‐COF‐Ni precursor was obtained.

TpDt‐COF before Ni immobilization was characterized by several techniques (Figure S2 to S5). The specific N_2_ sorption isotherms and the pore size distribution are presented in Figure S2a, b. The specific surface area (derived from the Brunnauer–Emmett–Teller theory, BET) reached 253 m^2^ g^−1^, with a pore size of about 1.2 nm, which is close to the theoretical one. The successful synthesis allowed the facile incorporation and immobilization of Ni ions into the backbone. The scanning electron microscopy (SEM) image and the X‐ray diffraction (XRD) pattern (Figure S2c, d) of TpDt‐COF indicate a flake‐like morphology and low crystallinity, consistent with the earlier reported COF.^[14]^ From the Fourier transform infrared (FT‐IR) spectra, the absence of aldehyde and amine groups stretching vibration in TpDt‐COF implies a complete condensation (Figure S3).[^14^, ^15^] As shown in the ^13^C solid‐state nuclear magnetic resonance (NMR) profile, the characteristic signals of C−N bond at ≈149 ppm, C=C bond at ≈108 ppm, and carbonyl carbon (C=O) at ≈184 ppm corroborated the formation of β‐ketone‐amine structure (Figure S4). The thermogravimetric analysis (TGA) showed the gradual decomposition of TpDt‐COF after 400 °C (Figure S5).

### Synthesis of the C‐TpDt‐Ni Catalyst

TpDt‐COF provides a unique structure to immobilize Ni−N_*x*_ functionalities; however, COFs notoriously suffer from low electric conductivity. Therefore, thermal annealing was used to carbonize the framework. The heat treatment of the TpDt‐Ni was conducted under inert gas (N_2_) and followed by an acid washing protocol (to remove formed exposed Ni particles). The pyrolysis temperature was changed from 800 °C, 900 °C, to 1000 °C to obtain a series of distinct carbonized C‐TpDt‐Ni as CO_2_RR catalysts. The synthesis protocol is shown in Figure 1a.

### Synthesis of Additional Reference Catalysts

Besides the C‐TpDt‐Ni catalysts, additional Ni‐N‐C materials were prepared as references. An additional COF‐derived catalyst was synthesized via an analogous protocol but using a different linker, para‐phenylene‐diamine (Pa, instead of Dt, see Figure S6a). Identical post‐treatments were done on this TpPa‐COF, and the pyrolysis temperature was selected to be 900 °C for the final catalyst, named C‐TpPa‐Ni‐900. The unpyrolyzed TpPa‐COF framework shows a surface area of 571 m^2^ g^−1^ and also high crystallinity. However, the BET surface area drops to 135 m^2^ g^−1^ after pyrolysis and acid washing (Figure S6, Table S1). Furthermore, a carbon nanotube supported Ni‐phthalocyanine (NiPc/CNT), and our previously studied polyaniline derived PANI‐Ni‐900 were involved in our experimental comparison (synthesis details are represented in the Supporting Information).

### Characterization

The morphology of the pyrolyzed C‐TpDt‐Ni was investigated using scanning electron microscopy (SEM) and transmission electron microscopy (TEM). As shown in Figure 1b, c and Figure S7, the nanosheets of TpDt‐COF fused to form porous particles, showing the co‐existence of micropores and mesopores. In addition, a small amount of Ni metal particles was observed in C‐TpDt‐Ni by low magnification TEM measurement (Figure S8). Considering those particles are encapsulated in an approximately 3 nm carbon shell (Figure S8b, unreachable in acid washing), they would only play a minor role during the electrolysis.[^10b^, ^16^] The BET surface area of the C‐TpDt‐Ni samples decreased to ≈190 m^2^ g^−1^ after pyrolysis/acid‐washing, caused by the structural changes during carbonization (pore‐collapse or sheet‐distortion, details are given in Figure S9 and Table S1).

HAADF‐STEM was applied to analyze the structure and surface composition of C‐TpDt‐Ni‐800 and C‐TpDt‐Ni‐900 samples. The STEM (Figure 1d, e and Figure S10a–c) images evidence graphitized carbon layers and directly proved the existence of atomically dispersed Ni sites (highlighted by red dashed circles) in both pyrolyzed samples. For the sample treated at 900 °C, the electron energy loss spectrum (EELS) was measured at one single Ni atom location, and a Ni‐N_4_‐C matrix‐like surrounding could be observed (Figure 1f).^[17]^ HAADF‐STEM images with other scale and corresponding energy‐dispersive X‐ray spectroscopy (EDS) mapping are shown in Figure 1g, h, proving Ni and N elements′ homogeneous distribution.

The XRD patterns of the catalysts are summarized in Figure S11. All samples exhibited a pronounced Bragg peak at around 26°, assigned to the (002) plane of graphitic carbon. For the C‐TpDt‐Ni samples, the peak at 26° became sharper with increasing pyrolysis temperature, suggesting an increasing graphitization degree. A weak diffraction peak at 44° is observed in the catalysts obtained at 900 and 1000 °C (C‐TpDt‐Ni‐900 and C‐TpDt‐Ni‐1000), which can be assigned to the (111) plane of Ni metal species. This stands in line with the TEM images of the samples (Figure S8a). As expected, the electronic conductivity of TpDt‐derived catalysts, measured using a 4‐probe sensing, increased with increasing temperature (Table S2). The chemical state and elemental composition of the catalyst surface were analyzed using X‐ray photoelectron spectroscopy (XPS), and the profiles are plotted in Figure S12 (Table S3–S5). The analysis of carbon 1s, nitrogen 1s spectra are given in the Supporting Information. In Figure S12c, the XPS profiles of the Ni2p_3/2_ core level range are presented, which indicate the chemical state and amount of Ni at the surface. Different groups were assigned according to their central 2p_3/2_ photoemission peak positions (Group 0: 852.6±0.2 eV, Ni^0^; Group I: 854.4±0.2 eV, Ni^+^‐like; Group II: 855.3±0.2 eV, Ni^2+^; and the satellite region: >856.5 eV).[^7b^, ^8^, ^18^] In the pristine TpDt‐Ni sample, the apparent satellite intensity suggests the prevalence of Ni in its +2 state.^[19]^ After pyrolysis, the intensity of Ni^0^ species slightly increased (see 900 °C), which can be attributed to the formed carbon‐encapsulated Ni nanoparticles (Figure S8). However, in all pyrolyzed samples, Ni^2+^ and Ni^+^‐like remained the dominant species. Due to the removal of all unencapsulated metallic Ni species by acid washing, we conclude that both the Ni^2+^ and Ni^+^‐like states in the pyrolyzed samples indicate the presence of single metal atom site Ni−N_*x*_ moieties, which are believed to serve as catalytic active sites. Accordingly, we estimate the Ni−N_*x*_ sites ratios (%_at._) on the surface via Equation 1 (Figure S13c) and extract the exposed SDs in combination with BET area‐weight surface [Eq. 2 and Figure S13d].

(1) $$x_{Ni-N_{x}}{\%}_{at.}=x_{{Ni}^{+}}{\%}_{at.}+x_{{Ni}^{2+}}{\%}_{at.}$$

(2) $${SD}_{Ni-N_{x}}=x_{Ni-N_{x}}{\%}_{at.}\times A_{BET}$$

Furthermore, the bulk Ni content of the catalysts was determined by inductively coupled plasma mass spectroscopy (ICP‐MS), and yielded 5.0 wt%, 7.6 wt%, and 0.8 wt% for C‐TpDt‐Ni‐800, C‐TpDt‐Ni‐900, and C‐TpDt‐Ni‐1000, respectively. Altogether, the TpDt‐Ni‐900 sample provides a higher SD than other carbonized COF counterparts (Figure S13).

### X‐ray Absorption Spectra

To explore the local molecular structure of the Ni−N_*x*_ motif, the catalysts were investigated using X‐ray absorption near‐edge structure (XANES) and extended X‐ray absorption fine structure (EXAFS) spectroscopies at Ni K‐edge coupled to a wavelet transformed EXAFS analysis. For reference, the measurements were carried out also on Ni foil, NiO, and NiPc.

**Figure 2:**
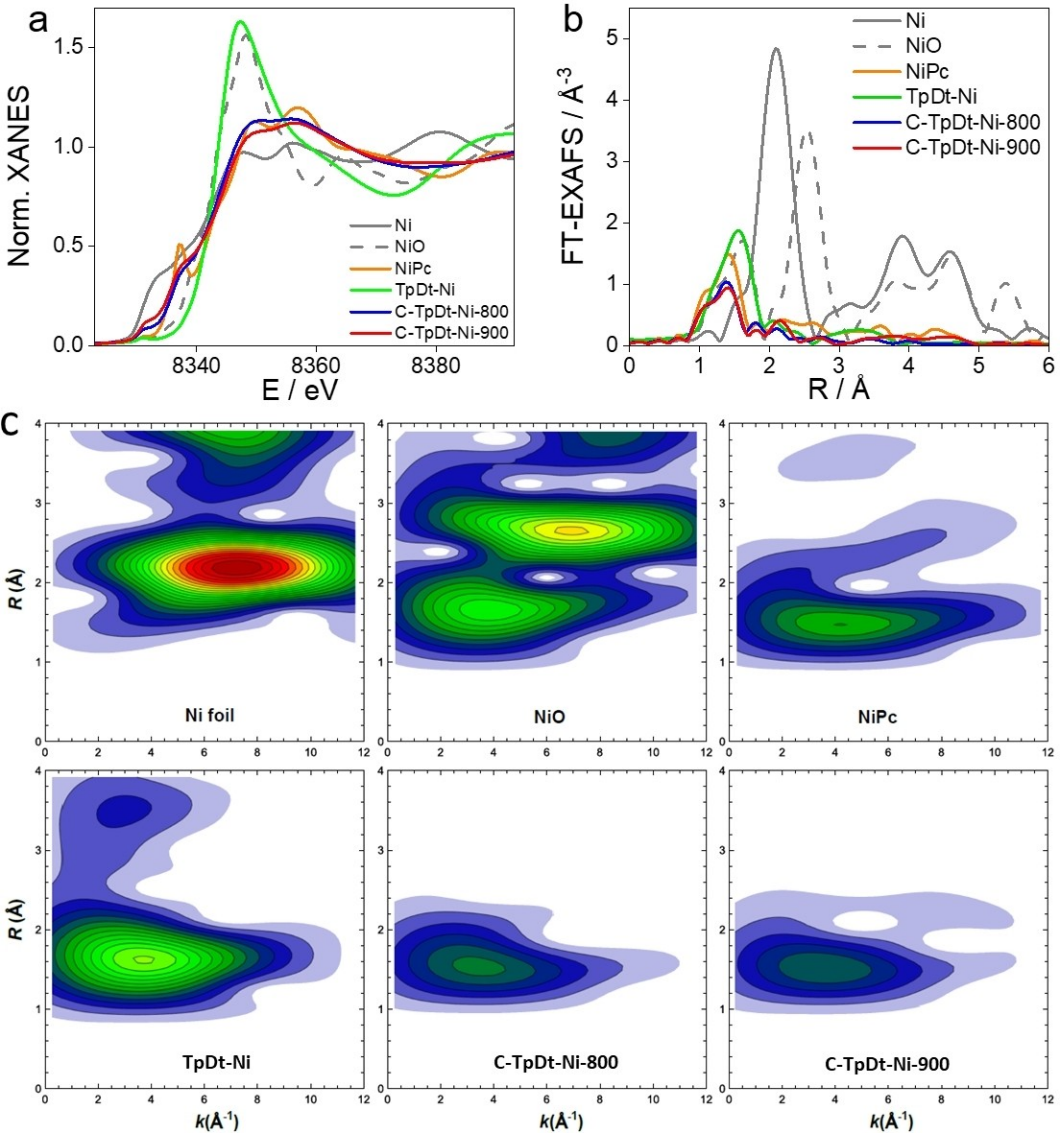
a) Normalized Ni K‐edge XANES spectra, b) absolute values of Fourier‐transformed (FT)‐EXAFS spectra, and c) absolute values of wavelet transformed (WT)‐EXAFS spectra for Ni foil, NiO, NiPc, TpDt‐Ni, C‐TpDt‐Ni‐800 and C‐TpDt‐Ni‐900. The structure parameters obtained from EXAFS data fitting are presented in Table S6.

The collected normalized Ni K‐edge XANES spectra are compared in Figure 2a. The significant difference between the spectra for unpyrolyzed (TpDt‐Ni) and pyrolyzed (C‐TpDt‐Ni‐800 and C‐TpDt‐Ni‐900) samples is evident immediately. The spectrum of the unpyrolyzed sample resembles that of rocksalt‐type NiO, suggesting a +2‐oxidation state and an octahedral coordination of Ni in this sample. On the other hand, the spectra for the pyrolyzed samples are more similar to the spectrum of the NiPc reference sample. In particular, the prominent feature at ca. 8337 eV is a fingerprint of a square‐planar Ni−N_4_ configuration with *D*4_h_ symmetry in porphyrin‐like structures located in di‐vacancy (DV).[^16^, ^20^] In the Ni‐based catalysts, this feature is also clearly present even though is reduced in intensity in comparison to NiPc. This is in agreement with previous reports in the literature,[^16^, ^21^] suggesting that the structure of the pyrolyzed catalysts is significantly disordered. The XANES spectra for samples pyrolyzed at 800 and 900 °C, respectively are quite similar. Still, one can note that for the sample pyrolyzed at 900 °C, the XANES spectrum is shifted slightly towards lower energies, e.g., closer to the metallic Ni spectrum, which could be an indication of the formation of metallic Ni clusters. All these findings are in line with the above‐described TEM and XPS analyses.

We performed linear combination fitting of the XANES spectra to quantify this trend by using spectra for metallic Ni foil and NiPc as references (Figure S14). Due to the significant differences between the local Ni structures in the catalyst samples and the reference samples, the linear combinations of reference spectra do not describe the XANES spectra for the catalysts perfectly. Still, one can estimate from such a fitting that the contribution of metallic Ni increases from ca. 15 % in the sample pyrolyzed at 800 °C to ca. 28 % in the sample pyrolyzed at 900 °C.

Fourier transforms (FT) of extracted EXAFS spectra (Figure S15) for Ni catalysts and the reference samples are shown in Figure 2b. For all catalyst samples, the EXAFS signal is dominated by the first coordination shell contribution (peak in FT‐EXAFS between 1 and 2 Å (phase‐uncorrected)), corresponding to the Ni bonding to low Z elements (e.g., oxygen, carbon, or nitrogen). However, one can note a difference in the main FT‐EXAFS peak position for the pyrolyzed and unpyrolyzed samples. In the former case, the maximum peak is located at lower *R* values, and it aligns well with the first shell peak (corresponding to Ni−N bond) in the reference spectrum for NiPc. For the unpyrolyzed sample, the maximum of the main FT‐EXAFS peak is shifted to slightly larger *R* values. This peak position and intensity are thus comparable to that of Ni−O contribution in the NiO spectrum.

In all FT‐EXAFS spectra, the weaker peaks at *R* values between ca. 2 and 4 Å are also observed. For visualization and interpretation of these contributions, it is convenient to use the Morlet wavelet transform (WT).^[22]^ WT‐EXAFS spectra for Ni catalysts and reference samples are shown in Figure 2c. First, for the unpyrolyzed TpDt‐Ni, it can be observed that the position of the main maximum in the WT‐EXAFS map (corresponding to the 1^st^ coordination shell), not only in *R*‐space but also in *k*‐space matches well to that of Ni−O contribution in the NiO reference spectrum, which are seen at a *R*‐value of ca. 1.6 Å, and *k* value of ca. 3.5 Å^−1^. Furthermore, the second WT‐EXAFS peak at a *R*‐value of ca. 3.4 Å has a maximum at a similar *k* value. From this, we conclude that this contribution at higher *R*‐values is also associated with photoelectron interactions with low Z elements (N, C, or O) and maybe even a result of multiple scattering events within the first coordination shell. The low amplitude of this feature and the absence of peaks at higher *R*‐values suggest a lack of long‐range order and a disordered local environment around Ni in the unpyrolyzed sample. Lack of contributions at higher *k*‐values confirms the absence of a significant amount of metallic Ni clusters in this sample. Nevertheless, one should note that caution is needed when interpreting the lack of distant coordination contribution as evidence for the single‐atom nature of the catalyst, and the presence of small amounts of disordered larger clusters cannot be completely ruled out.^[23]^

For the pyrolyzed C‐TpDt‐Ni samples, WT‐EXAFS is dominated by the maximum at *k*‐value ca. 3.5–4 Å^−1^ and *R*‐value ca. 1.5 Å, which aligns well with that of Ni−N contribution in NiPc. Significantly, upon increasing pyrolysis temperature, a second feature is gradually developed at *k* value ca. 8 Å^−1^ and *R*‐value ca. 2.2 Å, which strongly resembles the Ni−Ni contribution in metallic Ni. The WT‐EXAFS spectra thus reveal the formation of metallic clusters at higher pyrolysis temperatures.

## Electrochemical CO_2_RR Activity Evaluation: TOF vs. SD

The CO_2_RR performance of our studied COF‐derived Ni‐N‐C catalysts was assessed in a liquid phase three‐electrode H‐cell. The testing potential varied from −0.4 V_RHE_ to −0.9 V_RHE_, and each electrolysis condition was kept stationary for 15 min for gas analysis. CO and H_2_ contribute as the main products during the bulk reaction. No liquid products were detected after the bulk electrolysis. Overall, all our tested Ni‐based (together with all references) catalysts show their dominant selectivity to CO; however, they deliver deviated mass‐based activity.

**Figure 3:**
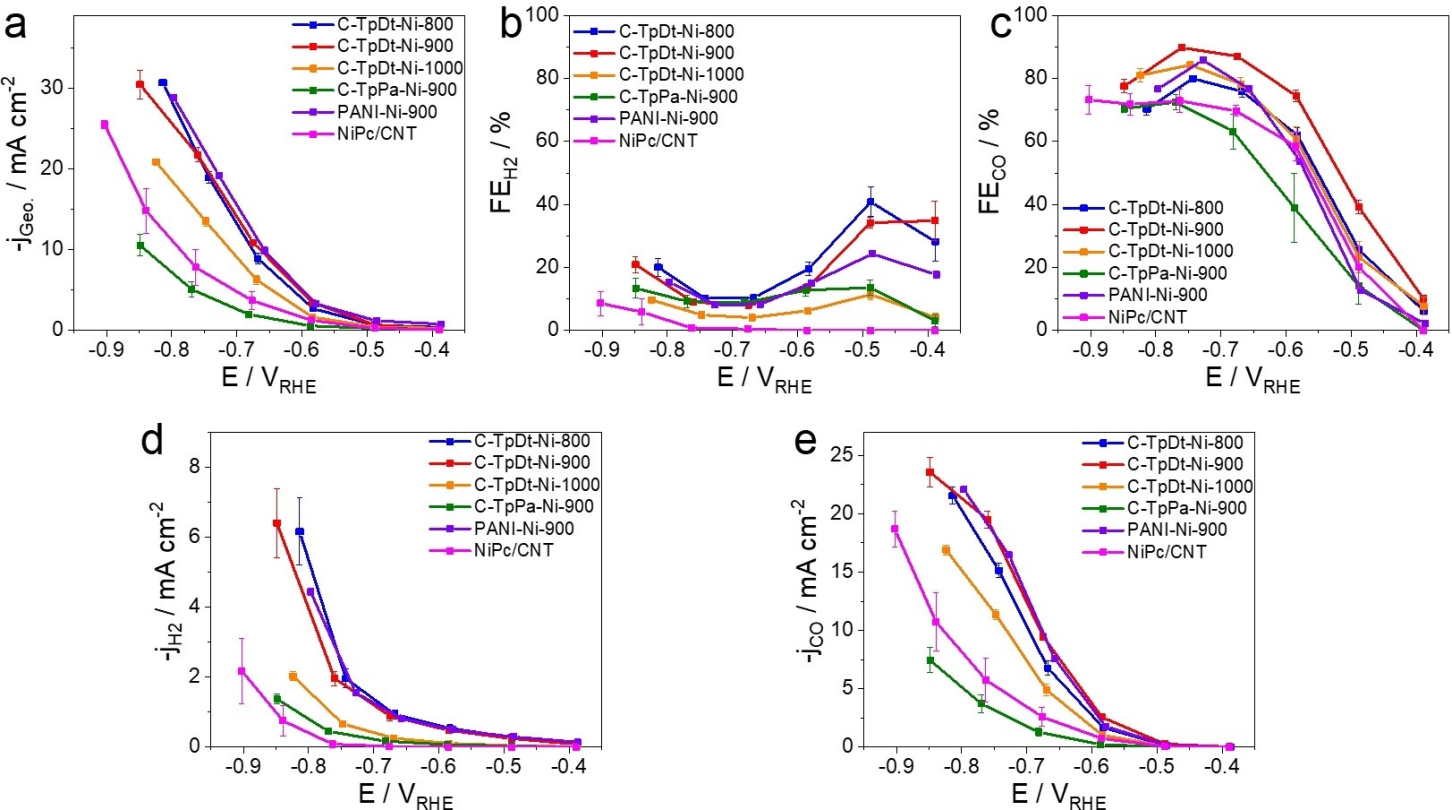
The electrochemical performance of various COF‐derived Ni‐N‐C and reference catalysts. a) Geometric current densities, b) Faradaic efficiency of H_2_, c) Faradaic efficiency of CO, d) geometric H_2_ partial current densities, and e) geometric CO partial current densities as functions of IR‐corrected potential. Calculations are described in Supporting Information (Eq S1–S4). The catalyst loading is 1 mg cm^−2^ on Freudenberg C2H23 carbon paper. The electrolyte is CO_2_‐saturated 0.5 M KHCO_3_ solution (pH 7.3). The product analysis is done after 15 min of each bulk electrolysis. Presented data is standard mean and deviation from 3 measurements.

The unpyrolyzed TpDt‐Ni shows negligible CO_2_RR reactivity, while the thermal treatment switches on the CO_2_ conversion to CO. The C‐TpDt‐Ni‐900 (pyrolyzed at 900 °C) sample reached over 90 % FE_CO_ at −0.7 V_RHE_, delivering about 22 mA cm^−2^ CO partial current density. The sample treated at 800 °C shows comparable but slightly lower performance. By contrast, the C‐TpDt‐Ni‐1000 delivers only 2/3 of the HER and CO_2_RR activity as the catalysts pyrolyzed at lower temperatures. All those outperform the C‐TpPa‐Ni‐900 one, prepared using the precursor with less nitrogen content (Figure 3).

The distinct CO_2_RR reactivity and selectivity values of the individual Ni‐N‐C catalysts are a sensitive function of, on the one hand, their molecular nature and intrinsic reactivity (TOF) and, on the other hand, their surface SD of catalytic active Ni−N_*x*_ moieties on the catalyst surface. In previous works, individual TOF and SD values remained convoluted and have not been properly disentangled, which is one reason why their accurate correlation to theoretical reactivity data has remained challenging and elusive. Here, we normalized the apparent CO activity to the SD values [from Eq. (2)] and obtained intrinsic kinetic TOF values [Eq. (S5)].

**Figure 4:**
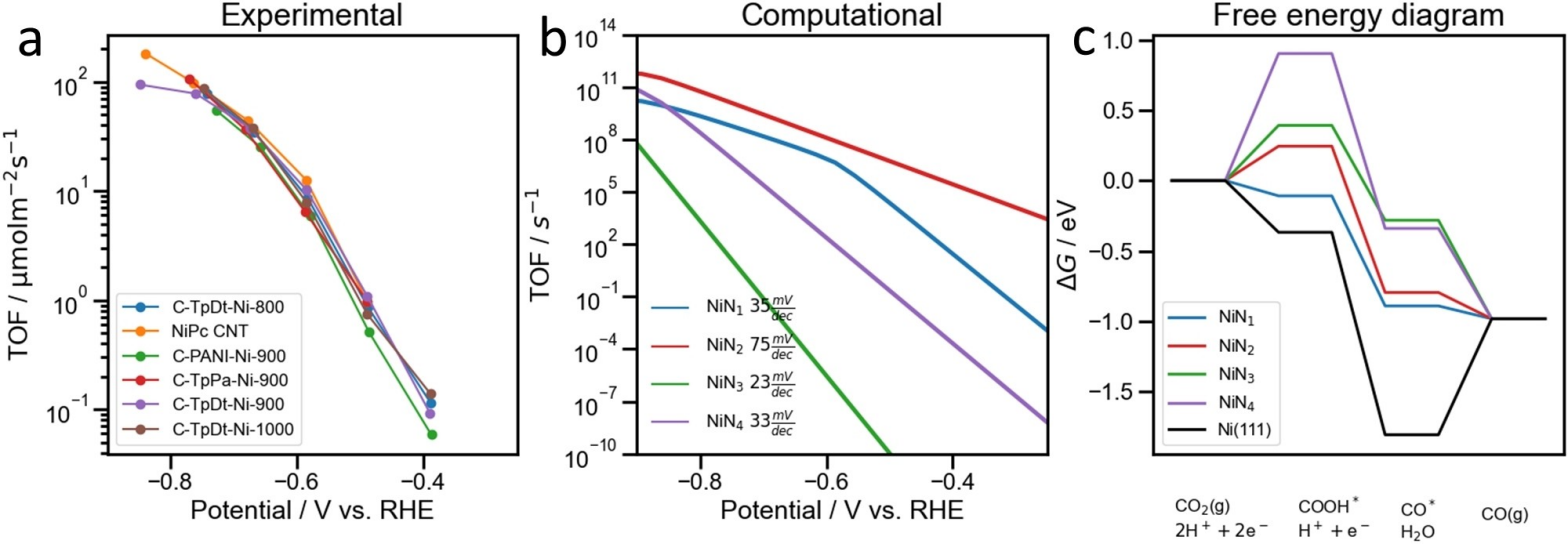
a) The experimentally derived CO turnover frequency (TOF) from −0.4 to −0.85 V_RHE_, normalized by Ni−N_*x*_ site density (SD). Apparent Tafel slopes are approximately 100 mV dec.^−1^ b) Theoretical TOF obtained from a micro‐kinetic model of the CO_2_(g) to CO(g) electro‐reduction process (calculation detailed in Supporting Information). c) The free energy diagram of CO_2_RR from CO_2_(g) to CO(g) on various DV‐Ni−N_*x*_ (*x*=1, 2, 3, 4) motifs and Ni(111) facet at −0.8 V_SHE_ in pH 4. The projected density of state (PDOS) plots of all studied Ni−N_*x*_ motifs are displayed in Figure S16.

The extracted experimental intrinsic CO‐specific TOFs of the various Ni‐N‐C catalysts from −0.4 to −0.85 V_RHE_ (kinetic region) are displayed in Figure 4a. Although this set of Ni‐N‐C catalysts were pyrolyzed at different temperatures during their preparation and displayed dissimilar FE values and partial current densities, after normalization of the apparent current densities using the extracted SD values, very similar intrinsic TOF trends ensued within. Furthermore, very similar TOF is observed on the molecular‐derived NiPc/CNT and the solid‐state PANI‐Ni reference catalysts. This finding suggests that the intrinsic catalytic behavior may be controlled by one dominant common site motif present in all materials.

To understand the TOF trends on the molecular scale, we separated the potential dependent TOF into two regions. From −0.4 to −0.75 V_RHE_, the CO production is primarily controlled by the applied electrode potential. Here, the nearly overlapping TOF‐potential trends of the tested catalysts imply a similar kinetic reaction barrier: the binding energy to the COOH* intermediate.^[24]^ DFT calculations show the trends on various simplified DV Ni−N_*x*_ (*x*=1, 2, 3, 4) motifs, and the Ni(111) facet. Displayed in Figure 4c, the nitrogen coordination number significantly affects the COOH* binding and leads to distinct theoretical TOF‐potential trend lines (Figure 4b). Instead, in experiments, all TOF values followed a similar trend as the CNT‐supported molecular NiPc (Figure 4a). Therefore, the experimentally observed TOF trends seem to originate predominantly from the fully embedded Ni−N_4_ coordination.

Furthermore, looking at larger cathodic potentials (beyond −0.7 V_RHE_, shown in Figure 3e) the C‐TpDt‐Ni‐900 electrocatalyst was the first to enter a CO_2_ mass transport‐limited reaction regime. By plotting the faradaic CO efficiency as a function of the current strength, the FE_CO_ of all catalysts appears to reach or pass through a plateau beyond 20 mA cm^−2^ (Figure S17a). We attribute this to in‐pore CO_2_ transfer limitations, where densely spaced active sites generate a high local pH. The in‐pores OH^−^ convert CO_2_ in equilibrium reactions to bicarbonate, causing rapid locally non‐electrochemical (acid‐base) CO_2_ depletion (scheme displayed in Figure S17b).

## Conclusion

In this contribution, we synthesized a set of novel TpDt‐COF‐derived Ni‐N‐C single metal atom site catalysts. Even though harsh synthetic steps such as pyrolysis and acid washing were performed, the Ni‐N‐Cs derived from TpDT‐COF at different temperatures exhibit similar surface areas, microporous structures, and comparable surface chemical composition, while their Ni−N_*x*_ site densities are largely different. This provides reliable materials basis to study the mass activity of catalysts regarding the two descriptors, intrinsic active site activity vs. number of active sites (TOF vs. SD).

Based on this family of Ni‐N‐C catalysts, we investigated reactivity trends and provided a new molecular understanding of the CO_2_ to CO reduction reactivity. What sets this work apart from earlier ones is that we deconvolute the usually reported apparent experimental CO_2_‐to‐CO geometric‐ or else mass‐based catalyst activity into two relevant descriptors, SD and TOF. Moreover, we correlated synthesis conditions with TOF values and compared experimental trends with computational DFT‐derived ones. Various characterization techniques (TEM, HAADF‐STEM, BET, XPS, XAS) identified and quantified the active Ni−N_*x*_ sites, while, in parallel, DFT‐derived Gibbs free energy diagram guided our understanding of the reaction kinetics from the atomic level.

We found that the apparent CO_2_‐to‐CO mass activities were highly dependent on the SD values, dominated by the synthesis details (i.e., annealing temperatures). By contrast, TOF was affected by synthesis conditions to a much lesser degree. When aiming at high apparent CO_2_‐to‐CO activities, it can be thus suggested to apply sp^2^‐N rich precursors, as seen for the used COF precursor, as this can largely enhance the amount of Ni ion coordinated in the final N‐doped carbon, thus increasing the SD. Later, the annealing temperature plays multiple roles. On the one hand, higher temperature improves the catalyst conductivity and accelerates the Ni−N_*x*_ moieties formation, showing to be positive for the catalytic performance. On the other hand, higher temperatures also reduce the amount of nitrogen within the carbon framework, resulting in a lower amount of formed Ni−N_*x*_ species. For the present materials, 900 °C turned out to be ideal pyrolysis temperature for optimizing the SD and conductivity of the catalysts. Further towards their intrinsic activity, the Tafel slope comparisons between experiment and theory suggested the presence and the predominant catalytic contribution of the DV Ni−N_4_ sites, evidenced in our absorption and photoemission studies.

In the high current density region, the TOF values saturated for the high‐SD catalysts, which is attributed to the local CO_2_ concentration depletion due to the high local OH^−^ concentration. As reported recently, using a rotating electrode or more acidic media could neutralize the surface pH, ensuring the CO_2_ transfer to the catalyst surface.^[25]^ Moreover, controlling the inner‐particle pore size (of the catalyst) and inter‐particle pore size (of the manufactured catalyst layer) tends to be a practical approach. To our hypothesis, the broader interfacial pores may accelerate the OH^−^ removal, maintaining the CO_2_ incoming at high current densities.

## Conflict of interest

The authors declare no competing financial interest.

1

## Supporting information

As a service to our authors and readers, this journal provides supporting information supplied by the authors. Such materials are peer reviewed and may be re‐organized for online delivery, but are not copy‐edited or typeset. Technical support issues arising from supporting information (other than missing files) should be addressed to the authors.

Supporting Information

## Data Availability

The data that support the findings of this study are available from the corresponding author upon reasonable request.
