# Supplementary material for: Covalent Organic Framework (COF) Derived Ni‐N‐C Catalysts for Electrochemical CO2 Reduction: Unraveling Fundamental Kinetic and Structural Parameters of the Active Sites
Source: Angew Chem Int Ed Engl. 2022 Feb 16;61(15):e202114707. doi: 10.1002/anie.202114707 (PMC9306911; doi:10.1002/anie.202114707)
Supplement: Supplementary file 1 — Supporting Information [file ANIE-61-0-s001.pdf]

Angewandte Chemie
Eine Zeitschrift der Gesellschaft Deutscher Chemiker

Supporting Information

# Covalent Organic Framework (COF) Derived Ni-N-C Catalysts for Electrochemical $CO_2$ Reduction: Unraveling Fundamental Kinetic and Structural Parameters of the Active Sites

*C. Li, W. Ju*, S. Vijay, J. Timoshenko, K. Mou, D. A. Cullen, J. Yang, X. Wang, P. Pachfule, S. Brückner, H. S. Jeon, F. T. Haase, S.-C. Tsang, C. Rettenmaier, K. Chan, B. R. Cuenya, A. Thomas*, P. Strasser**

Supporting Information

# Covalent Organic Framework (COF) derived Ni-N-C Catalysts for Electrochemical $CO_2$ Reduction:

## Unraveling Fundamental Kinetic and Structural Parameters of the Active Sites

Changxia Li[+ a], Wen Ju[+*b], Sudarshan Vijay[c], Janis Timoshenko[d], Kaiwen Mou[b], David A. Cullen[e], Jin Yang[a], Xingli Wang[b], Pradip Pachfule[a], Sven Brückner[b], Hyo Sang Jeon[d], Felix T. Haase[d], Sze-Chun Tsang[C], Clara Rettenmaier[d], Karen Chan[c], Beatriz Roldan Cuenya[d], Arne Thomas*[a], Peter Strasser*[b]

[+] These authors contributed equally to this work.

Affiliation

[a] *Department of Chemistry, Division of Functional Materials, Technical University Berlin, Berlin 10623, Germany*

[b] *Department of Chemistry, Chemical Engineering Division, Technical University Berlin, Berlin 10623, Germany*

[c] *CatTheory, Department of Physics, Technical University of Denmark, 2800 Kongens Lyngby, Denmark*

[d] *Interface Science Department, Fritz-Haber Institute of Max-Planck Society, Berlin 14195, Germany*

[e] *Center for Nanophase Materials Sciences, Oak Ridge National Laboratory, Oak Ridge, TN, USA*

# Contents


**Synthesis protocol of the C-TpDt-Ni- family catalysts** ... 3

*Synthesis of TpDt-COF* ... 3

*Synthesis of TpDt-Ni* ... 3

*Synthesis of pyrolyzed C-TpDt-Ni* ... 4

**Synthesis of reference Ni-N-C catalysts** ... 5

*Synthesis of C-TpPa-COF-900* ... 5

*Synthesis of NiPc/CNT* ... 5

*Synthesis of PANI-Ni-900* ... 6

**Characterization details** ... 7

*XRD, TGA, BET, ICP-OES, FT-IR, SEM, TEM, and HAADT-STEM* ... 7

*X-ray Photoelectron Spectra* ... 7

*X-ray absorption Spectra* ... 8

**Electrochemical performance screening** ... 9

*Electrode preparation* ... 9

*Electrochemical measurement* ... 9

*Cathode potential* ... 10

*Product analysis* ... 11

**Computational methods** ... 13

**Supplementary figures and tables** ... 14

*Characterization of TpDt-COF* ... 14

*Synthesis and characterization of TpPa-COF* ... 17

*Characterization of C-TpDt-Ni-family candidates* ... 18

*XRD patterns of all $CO_2$RR catalysts* ... 21

*XPS analysis* ... 22

*XAS analysis* ... 27

**Reference** ... 33

# Synthesis protocol of the C-TpDt-Ni- family catalysts

## Chemicals

All the chemicals were used without further purification. The 3,5-diamino-1,2,4-triazole (Dt), paraphenylenediamine (Pa), mesitylene (1,3,5-trimethylbenzene, > 98%), Ni phthalocyanine (NiPc, > 95%) and $Ni(NO_3)_2$ $6H_2O$ were purchased from Sigma Aldrich Chemicals. 1,4-dioxane (99.9 %), methanol (Carl Roth, ≥ 99%), acetone (≥ 99.5%), N,N-dimethylacetamide (DMA) and N,N-dimethylformamide (DMF) (≥ 99.9%) were purchased from Carl Roth.

## Protocol

### *Synthesis of Tp*

Tp was synthesized following our previous literature approach.[1] 15.1 g hexamethylenetetramine, 6.0 g phloroglucinol, and 90 mL trifluoroacetic acid were refluxed under $N_2$ at 100 °C for 2.5 h. 150 mL of 3M HCl was added slowly and the solution was heated at 100 °C for another 1 h. After cooling down, the solution was filtered through Celite and extracted with 350 mL dichloromethane. Then, the solution was evaporated under reduced pressure to afford 2.4 g of an off-white powder. Purification was carried out by sublimation.

### *Synthesis of TpDt-COF*

1,3,5-triformylphloroglucinol (90 mg, 0.42 mmol), 3,5-diamino-1,2,4-triazole (45 mg, 0.45 mmol) and 1.5 mL dioxane were put into a Pyrex tube and the mixture was sonicated for 5 min. 0.5 mL DMA and 1.5 mL mesitylene were added to the above solution and sonicated for 5 min. Following this, 0.5 mL of 6 M aqueous acetic acid was added. This mixture was sonicated for another 10 minutes to yield a homogenous dispersion. The tube was then flash frozen in a liquid nitrogen bath and degassed by three freeze-pump-thaw cycles. The tube was sealed and then placed in an oven at 120 °C for 3 days. After the mixture was cooled to room temperature, the reddish brown precipitate was collected and washed with hot DMF, methanol and acetone. The powder collected was then dried at 80 °C under vacuum for 12 hours to obtain a deep red colored powder (81%, isolated yield).

### *Synthesis of TpDt-Ni*

100 mg TpDt-COF was dispersed in 20 mL of $Ni(NO_3)_2$ $6H_2O$ (2M) aqueous solution and sonicated for 30 min and stirred for 10 h. The impregnated TpDt-COF was then separated by vacuum filtration and washed

with distilled water four times to remove weakly adsorbed ions. After drying at 80 $^{o}$C, the TpDt-Ni was obtained.

***Synthesis of pyrolyzed C-TpDt-Ni***

The TpDt-Ni was placed in a tube furnace and annealed at 900 °C (800 and 1000 °C) for 2 h under $N_2$ atmosphere (80 sccm) at a heating rate of 5.0 °C min$^{-1}$. The carbonized COF was dispersed in aqueous HCl (ca. 1 M) and stirred for 1 day. The leached sample was collected and washed with DI water until the pH value was close to neutral. The obtained materials were denoted as C-TpDt-Ni-T (where T represents the temperature).

## Synthesis of reference Ni-N-C catalysts

### *Synthesis of C-TpPa-COF-900*

### *Synthesis of TpPa-COF*

1,3,5-triformylphloroglucinol (63 mg, 0.3 mmol), paraphenylenediamine (48 mg, 0.45 mmol), 1.5 mL of mesitylene, 1.5 mL of 1,4-dioxane, 0.5 mL of 3 M aqueous acetic acid were placed into a Pyrex tube and the mixture was sonicated for 20 min to obtain a homogenous dispersion. The tube was then flash frozen in a liquid nitrogen bath and degassed by three freeze-pump-thaw cycles. The tube was sealed off and then placed in an oven at 120 °C for 3 days. After the mixture was cooled to room temperature, the red precipitate was collected and washed with THF and acetone. The powder collected was then dried at 80 °C under vacuum for 12 hours.

### *Synthesis of C-TpPa-Ni-900*

100 mg TpPa-COF was dispersed in 20 mL of $Ni(NO_3)_2\ 6H_2O$ (2M) aqueous solution and sonicated for 30 min and stirred for 10 h. The impregnated TpPa-COF was then separated by vacuum filtration and washed with distilled water four times to remove weakly adsorbed ions. After drying at 80 $^oC$, the TpPa-Ni was obtained. The TpPa-Ni was then placed in tube furnace and annealed at 900 °C for 2 h under $N_2$ atmosphere (80 sccm) at a heating rate of 5.0 °C $min^{-1}$. The carbonized COF was dispersed in aqueous HCl (ca. 1 M) and stirred for 1 day. The leached sample was collected and washed with DI water until a pH value close to neutral.

### *Synthesis of NiPc/CNT*

NiPc/CNT was synthesized following the analogous protocol reported in the literature.[2] 1 mg NiPc was mixed with 30 mg MWCNTs (multi wall carbon nanotubes) in 30 mL DMF solution and kept stirred for 24 hours. The suspension color turned from the initial violet to transparent. The final suspension was dried to obtain the NiPc/CNT catalyst.

*Synthesis of PANI-Ni-900*

PANI-Ni-900 was synthesized following our previous literature approach.[3] 3 ml of aniline, 5 g $NiCl_2 \cdot 6H_2O$ and 5 g ammonium persulfate (APS, $(NH_4)_2S_2O_8$) was added to 0.5 L of 1 M HCl and stirred for 1 hour. Then, the suspension was stirred for 48 hours along with 0.4 g of dispersed activated Ketjen 600 carbon black support (washed in HCl for purification and $HNO_3$ for oxygen doping). Afterwards, the suspension was dried at 95 °C for 24 hours. After drying, the solid mixture was ball-milled with $ZrO_2$ balls for 20 min. The pyrolysis is carried in a furnace with a ramp of 30°C $min^{-1}$ to 900 °C and kept at this temperature for 1 hour, in $N_2$ condition, and followed by acid washing steps (2M $H_2SO_4$ at 90 °C for overnight) to remove the excess Ni particles. In our synthesis, 4 times heat treatment (HT) and 3 times acid washing was performed by turn, and the catalyst is obtained after the 4$^{th}$ pyrolysis.

# Characterization details

## *XRD, TGA, BET, ICP-OES, FT-IR, SEM, TEM, and HAADT-STEM*

X-ray powder diffraction (XRD) patterns were performed on a Bruker D8 Advance instrument with Cu Kα radiation (λ=1.54 Å) operating at 40 kV and 40 mA. XRD patterns were collected in the range of 2°–60° at a scanning speed of 2° $min^{-1}$. Thermo-gravimetric analyses (TGA) were conducted using a Mettler Toledo TGA/DSC1 Star System analyzer at a heating rate of 10 °C $min^{-1}$ under $N_2$ atmosphere. $N_2$ sorption measurements was carried out using a Quantachrome Quadrasorb SI instrument with degassing temperature of 200 °C for 6 h for carbon samples and 120 °C for 12 h for COFs samples before the measurement. The specific surface areas were calculated by using Brunauer-Emmett-Teller (BET) calculations. The pore size distributions of COF and carbon samples were obtained from the adsorption branch of isotherms by the quenched solid density functional theory (QSDFT) and non-localized density functional theory (NLDFT) model, respectively. Bulk metal contents were measured with Varian 715-ES ICP-OES. For this, the samples were previously solved in aqua-regia and treated using microwave. Fourier transform infrared spectroscopy (FT-IR) analyses were carried out on Varian 640IR spectrometer equipped with an ATR cell. Scanning electron microscope (SEM) was measured using Gemini SEM 500 low vacuum high-resolution SEM. Transmission electron microscope (TEM) was performed using FEI Tecnai $G^2$ 20 S-TWIN electron microscope with an operating voltage of 200 kV. The conductivity was measured with Yokogawa GS610 Sourcemeter unit in galvanostatic mode between -2 mA and +2 mA in 0.2 mA steps. Aberration-corrected STEM images were recorded by using a high-angle annular dark-field (HAADF) detector equipped with a 54-200 mrad collection semi-angle at Oak Ridge National Laboratory.

## *X-ray Photoelectron Spectra*

XPS was performed on a K-Alpha X-ray photoelectron spectrometer system (Thermo Scientific) with Hemispheric 180° dual-focus analyzer with 128-channel detector. X-ray monochromator was microfocused Al Kα radiation. The samples were pasted and pressed onto the sample holder using carbon tapes for measurement.

*X-ray absorption Spectra*

XAS measurements at Ni K-edge (8333 eV) were performed at P64 beamline at PETRA-III synchrotron radiation facility (Hamburg). Measurements were performed in transmission mode. Intensities of incident radiation and transmitted radiation were measured with ionization chamber detector $I_0$ and $I_1$ filled with pure $N_2$. For data alignment, Ni foil's XAS spectrum was acquired in transmission mode simultaneously with the spectra for Ni samples. $I_2$ ionization chamber used for such reference measurements was also filled with pure $N_2$. Si (111) monochromator was used for energy selection. All measurements were performed in air at room temperature. ATHENA software was used for data alignment, normalization, and XAS spectra extraction.

# Electrochemical performance screening

## *Electrode preparation*

Carbon paper (1 cm × 2.5 cm, Freudenberg C2H23) was sonicated in ethanol and deionized water for 15 min and dried as the electrode substrate. The catalyst ink is prepared using 4.0 mg catalyst mixed with 60 µL Nafion solution (5% in ethanol, Sigma-Aldrich), 200 µL isopropanol, and 200 µL DI water. After 15 min sonification, the ink was deposited on the micro-porous-layer side of carbon paper to achieve an area of 1 $cm^2$ with catalysts loading of 1 mg $cm^{-2}$.

## *Electrochemical measurement*

The $CO_2$RR performance screening was carried out in a regular 3-electrode H-cell, divided by a Nafion N117 membrane. The working electrode was the catalysts-coated carbon paper mentioned above, and a Pt mesh was deployed as the counter electrodes. A leak-free Ag/AgCl electrode was used as the reference. Electrochemical experiments were performed in $CO_2$ purged 0.5 M $KHCO_3$ ($CO_2$ flow rate: 30 ml $min^{-1}$, pH: 7.3). The current density ($j$) was normalized to the working electrode's geometrical area (1 $cm^2$).

*Cathode potential*

The working potential is controlled by the Biologic SP-300 potentiostat against the Ag/AgCl reference electrode. Before the bulk electrolysis, the ohmic resistance between cathode and reference electrode was measured using PEIS (potentiostatic electrochemical impedance spectroscopy) module at -1.0 V vs. Ag/AgCl Ref, and the frequency was set from 100 k Hz to 1 Hz. Subseqently, 50% of the ohmic drop was automatically corrected, and the other half was corrected manually. All potentials were rescaled to reversible hydrogen electrode (RHE) by Eq. S1.

$$E_{IR-free} = E_{vs.\ Ref} + E_{Ref\ vs.\ NHE} + 0.059 * pH + I * R * 50\% \qquad \text{Eq. S1}$$

| | | |
|---|---|---|
| $E_{IR-free}$: | IR corrected cathode potential against RHE | / $V_{RHE}$ |
| $E_{vs.\ Ref}$: | Applied potential against the reference electrode | / V |
| $E_{Ref\ vs.\ RHE}$: | Reference electrode potential measured against NHE | / V |
| $pH$: | pH-value of the electrolyte | |
| $I$: | Total current of the experiment (absolute value) | / A |
| $R$: | Ohmic resistance between cathode and reference electrode | / Ω |

*Product analysis*

A Shimadzu 2014 on-line GC is utilized for product quantification. The gas stream is separated by the Hayesep Q + R columns and then analyzed by the TCD (Thermo Conductivity Detector) and FID (Flame Ionization Detector). The TCD detects the volume percentage ($\%_{VOL}$) of the $H_2$ product, and the FID measures the CO after being methanized. On the all Ni-N-C type catalyst, no liquid product is found after the electrolysis. Calculations of the production rate (Eq. S2), partial current density (Eq. S3), and faradaic efficiency (Eq. S4) are given below. The calculation of the TOF is given in (Eq. S5).

$$\dot{n}_{Product} = \frac{\dot{V}_{Total} \times C_{Product}}{A \times V_{MOL}} \qquad \text{Eq. S2}$$

| | | |
|---|---|---|
| $\dot{n}_{Product}$: | geometric reaction rate of each product | / mol cm$^{-2}$ s$^{-1}$ |
| $\dot{V}_{Total}$ : | Exhaust stream flow rate | / mL s$^{-1}$ |
| $C_{Product}$: | product concentration (volumetric ratio) detected by GC | / $\%_{VOL}$ |
| $A$: | geometric area of the electrode | / cm$^2$ |
| $V_{MOL}$: | volume of gas per mole at ATM | / mL mol$^{-1}$ |

$$j_{Product} = \dot{n}_{Product} \times F \times z \qquad \text{Eq. S3}$$

| | | |
|---|---|---|
| $j_{Product}$: | partial current density of each product | / A cm$^{-2}$ |
| $F$: | faradaic constant | / C mol$^{-1}$ |
| $z$: | charge transfer per product molecule | |

$$FE_{Product} = \frac{j_{Product}}{j_{Total}} \times 100\% \qquad \text{Eq. S4}$$

| | | |
|---|---|---|
| $FE_{Product}$: | faradaic efficiency of each product | / % |
| $j_{Total}$: | total current density | / A cm$^{-2}$ |

$$TOF = \frac{\dot{n}_{CO}}{M_{loading} \times x_{Ni-Nx}\%_{at.} \times A_{BET}}$$ Eq. S5

| | | |
|---|---|---|
| $TOF$: | CO Turnover Frequency normalized by active Ni-$N_x$ area | / mol $m^{-2}$ $s^{-1}$ |
| $\dot{n}_{CO}$: | geometric CO reaction rate | / mol $cm^{-2}$ $s^{-1}$ |
| $M_{loading}$: | catalyst loading | / mg $cm^{-2}$ |
| $A_{BET}$: | specific $N_2$ adsorption area measured using BET | / $m^2$ $mg^{-1}$ |
| $x_{N-Nx}\%_{at.}$: | atomic ratio of total exposed Ni-$N_x$ on surface | / $\%_{at.}$ |

# Computational methods

Density functional theory calculations were performed using Vienna Ab-initio Software Package (VASP).[4] Core electrons were described using Projector Augmented Waves (PAW) potentials.[5] Valence electrons were described using plane-waves with kinetic energy up to 500eV. Gaussian smearing with a width of 0.1eV was used. The RPBE[6] functional was used for all calculations. All calculations were run with spin-polarization.

Structures were prepared using the Atomic Simulation Environment (ASE).[7] The lattice constant of graphene was optimized using a 12x12x12 Monkhorst-Pack *k*-point mesh grid.[8] A 3×3 single-layer graphene structure was made with the obtained lattice parameter. All structures were then treated with a 4×4×1 Monkhorst−Pack k-point mesh with at least 10 Å of vacuum. Depending on the vacancy type, carbon atoms in the graphene structure were replaced by nitrogen and Nickel atoms. The structure obtained after creating vacancies and doping was subjected to an optimization of positions before adding an adsorbate to the unit cell. All geometries are optimized until forces are less than 0.025 eV $Å^{-1}$. The density of states were obtained by using Gaussian smearing of 0.1 eV.

The computational hydrogen electrode (CHE)[9] was used to determine reaction energies as a function of potential for reactions with an electron in the reactant or product. The chemical potential of the proton and electron is related to that of H2 at 0 V vs RHE using:

$$\mu_{\mathrm{H}^+} + \mu_{\mathrm{e}^-} = \frac{1}{2}\mu_{\mathrm{H}_2} \qquad \text{Eq. S6}$$

Microkinetic modelling was performed using CatMAP.[10] The rate of a given elementary step was:

$$\mathrm{rate} = k_+ \Pi\theta_i \Pi p_j - k_- \Pi\theta_i \Pi p_j \qquad \text{Eq. S7}$$

where $+$ indicates the forward reaction and – the reverse reaction. The rate constants can be given as $k_+ = \exp\left(-\frac{G_{a,+}}{k_B T}\right)$ and $k_- = \exp\left(-\frac{G_{a,-}}{k_B T}\right)$. In the absence of electrochemical barriers, the free energy is used, which is given as $\Delta G = \Delta G^o + neU + \Delta G_{\mathrm{field}}$, where $\Delta G^o$ is the free energy for the reaction at the potential of zero charge (pzc), $n$ is the number of proton-electron pairs transferred and $\Delta G_{\mathrm{field}}$ is the dipole-field contribution.

A multi-precision Newton root finding algorithm was used to determine the steady-staterates and coverages. A decimal precision of 100 along with a convergence tolerance value of $10^{-25}$ was used.

# Supplementary figures and tables

## *Characterization of TpDt-COF*

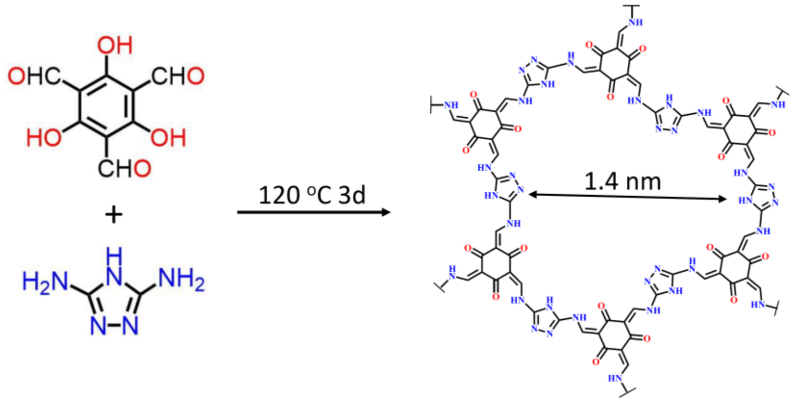


Figure S1. Schematic representation of the synthesis of TpDt-COF.

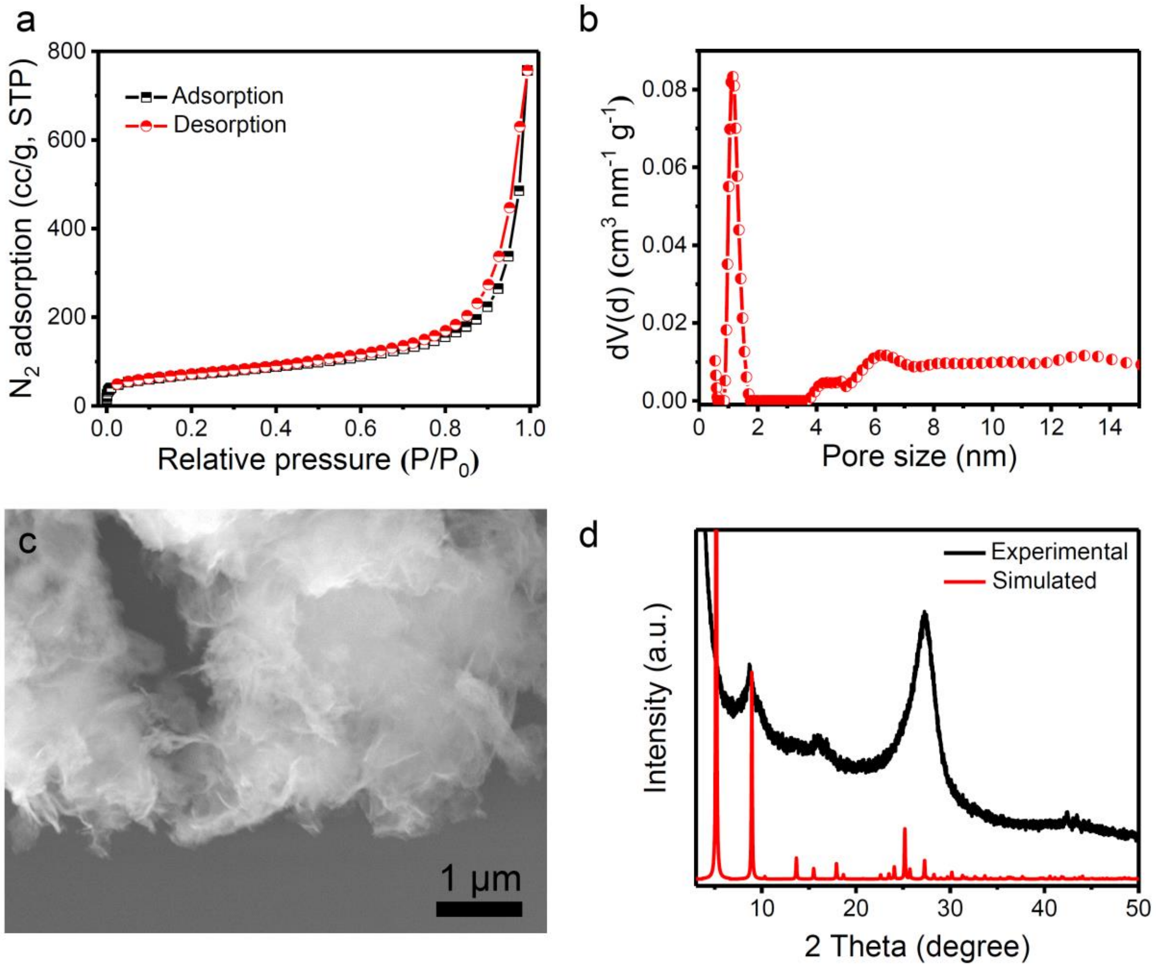


Figure S2. (a) $N_2$ absorption-desorption isotherms, (b) pore size distribution, (c) SEM image and (d) XRD pattern of TpDt-COF.

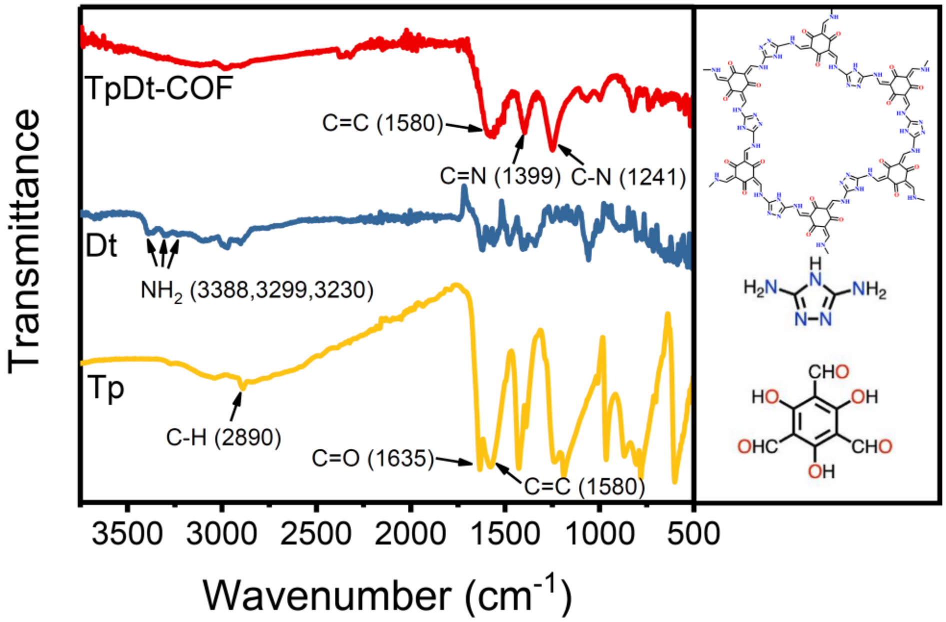


Figure S3. FT-IR spectroscopy of TpDt-COF, Tp and Dt.

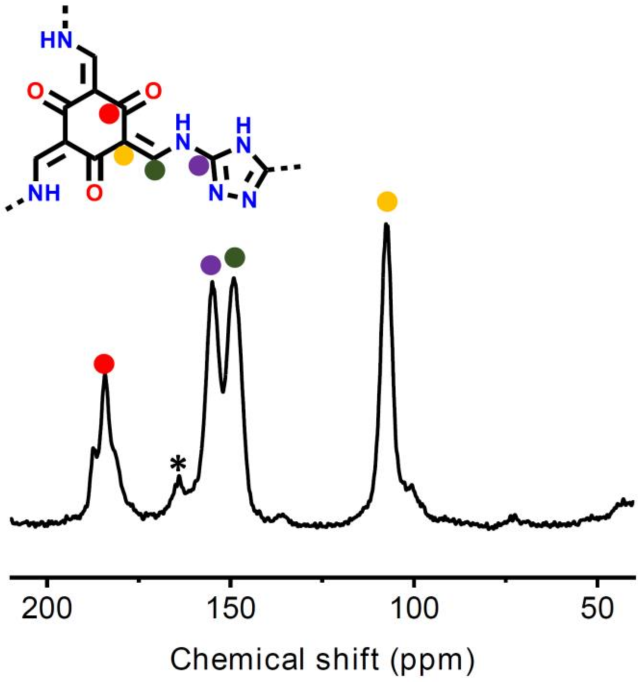


Figure S4. $^{13}C$ solid state NMR of TpDt-COF. *- corresponds to the solvent DMF.

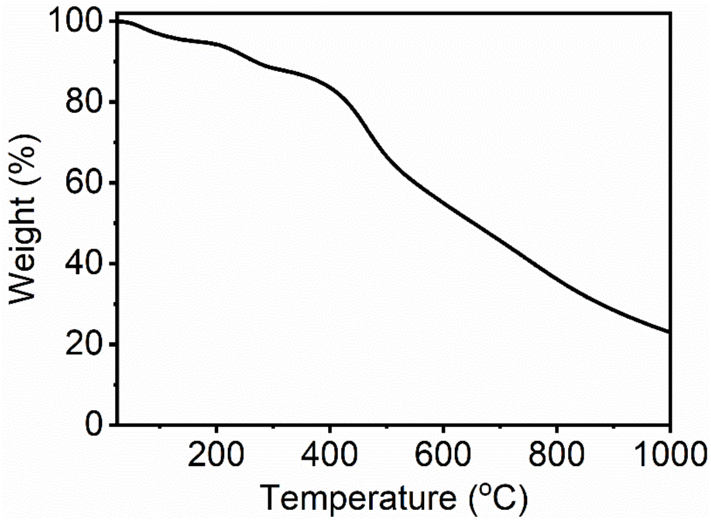


Figure S5. The TGA curve of TpDt-COF.

## *Synthesis and characterization of TpPa-COF*

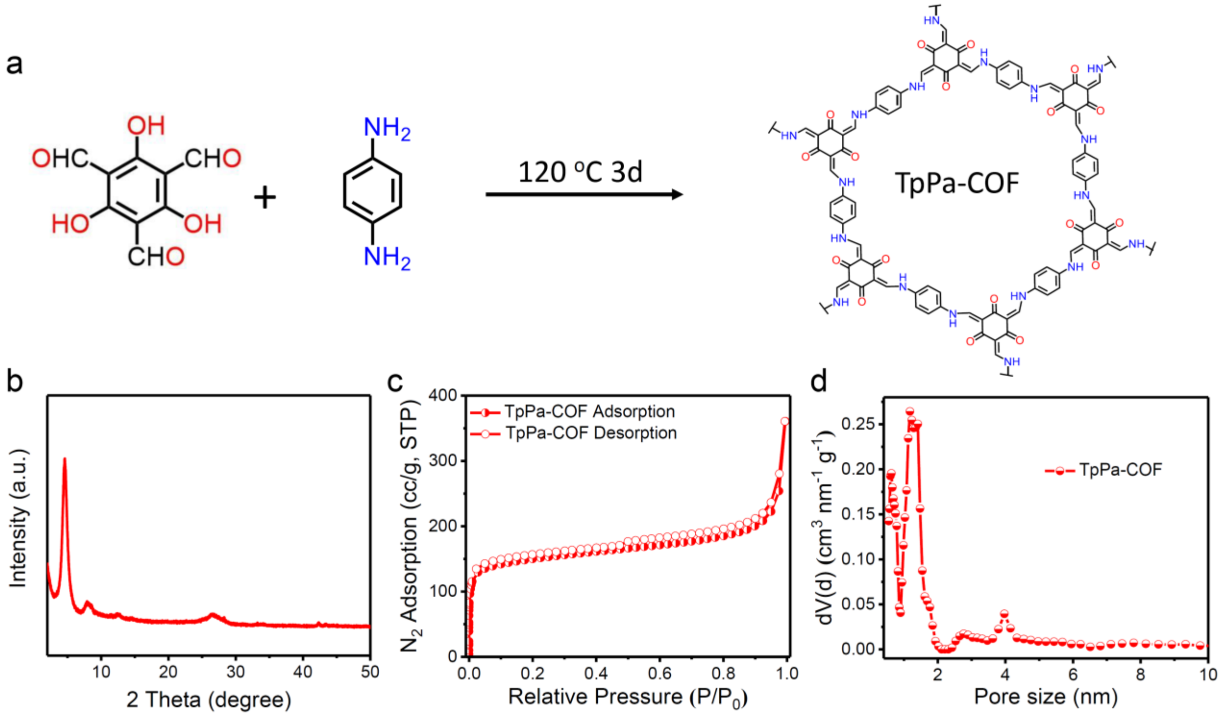


Figure S6. (a) Schematic representation of the synthesis of TpPa-COF. (b) XRD pattern, (c) $N_2$ absorption-desorption isotherms and (d) pore size distribution of TpPa-COF.

*Characterization of C-TpDt-Ni-family candidates*

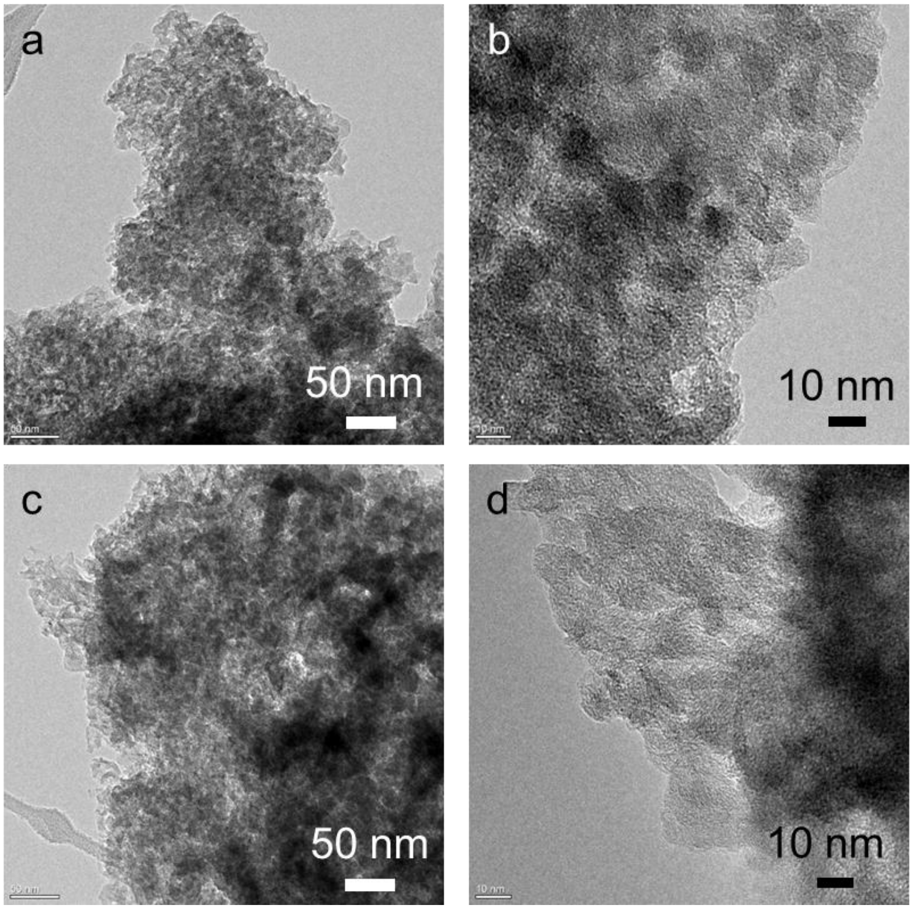


Figure S7. TEM images of (a, b) C-TpDt-Ni-800 and (c, d) C-TpDt-Ni-1000.

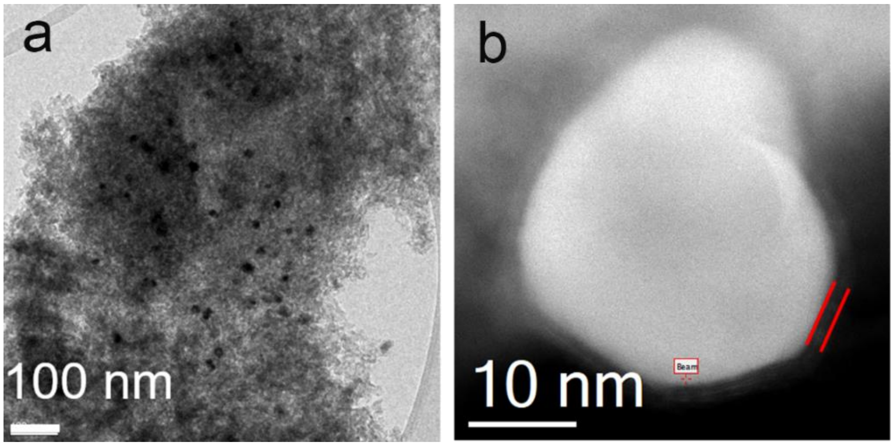


Figure S8. (a) Low magnification TEM image and (b) HAADF-STEM image of C-TpDt-Ni-900.

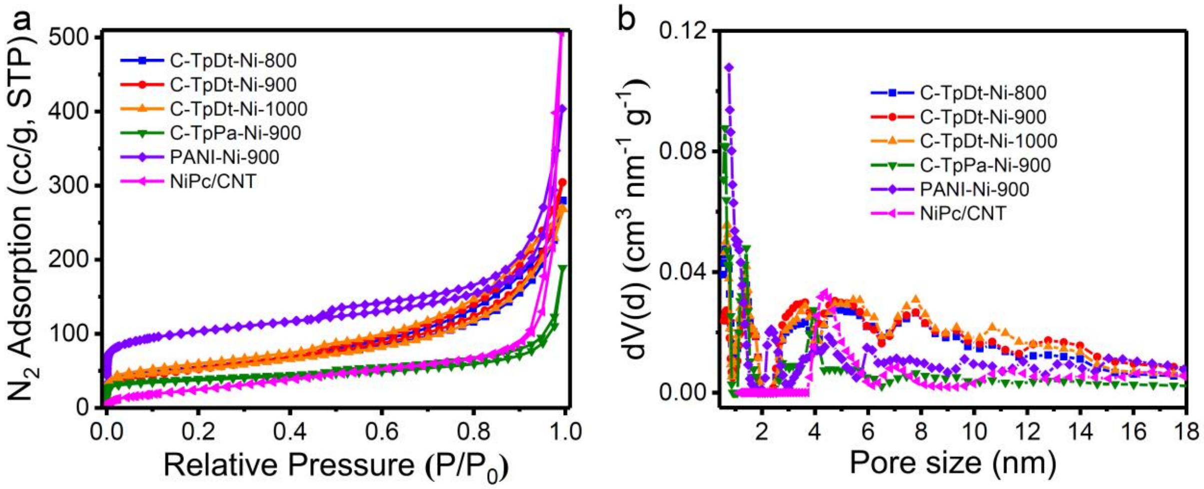


Figure S9. (a) $N_2$ absorption-desorption isotherms and (b) pore size distributions of C-TpDt-Ni-800, C-TpDt-Ni-900, C-TpDt-Ni-1000, C-TpPa-Ni-900, PANI-Ni-900, and NiPc/CNT.

**Table S1.** The BET specific surface areas and pore volume of all the samples involved.

| Samples | Specific surface area ($m^2\ g^{-1}$) | Pore volume ($cm^3\ g^{-1}$) |
|---|---|---|
| TpDt-COF | 253 | 0.597 |
| TpPa-COF | 571 | 0.369 |
| C-TpDt-Ni-800 | 192 | 0.335 |
| C-TpDt-Ni-900 | 188 | 0.380 |
| C-TpDt-Ni-1000 | 184 | 0.359 |
| C-TpPa-Ni-900 | 135 | 0.162 |
| PANI-Ni-900 | 414 | 0.413 |
| NiPc/CNT | 85 | 0.296 |

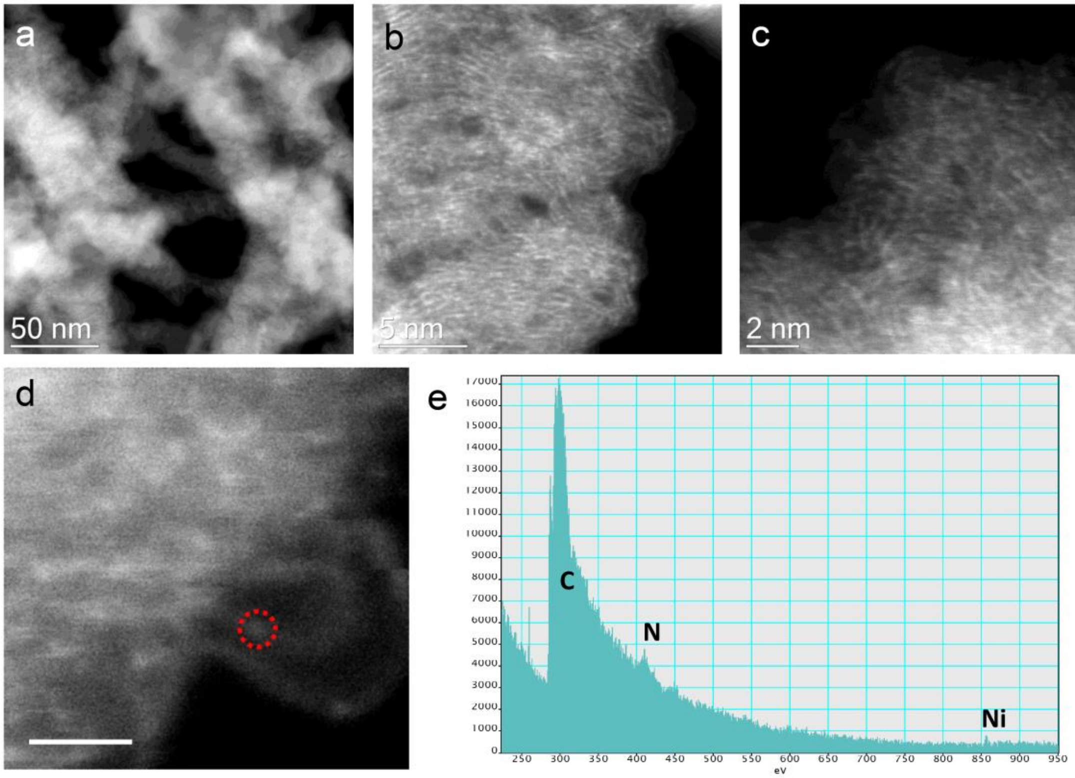


Figure S10. (a-d) HAADF-STEM images of C-TpDt-Ni-800 and (e) the corresponding EELS spectrum acquired at Ni single atom region circled in red from S10d.

**Table S2.** Conductivity of TpDt-COF, C-TpDt-Ni-800, C-TpDt-Ni-900, C-TpDt-Ni-1000.

| Samples | Conductivity (S $cm^{-1}$) |
|---|---|
| TpDt-COF | * |
| C-TpDt-Ni-800 | 0.24 |
| C-TpDt-Ni-900 | 1.98 |
| C-TpDt-Ni-1000 | 3.45 |

The power samples were pressed between steel cylinders in a PE-die.

* Resistance is too high, out of measurement range.

*XRD patterns of all $CO_2$RR catalysts*

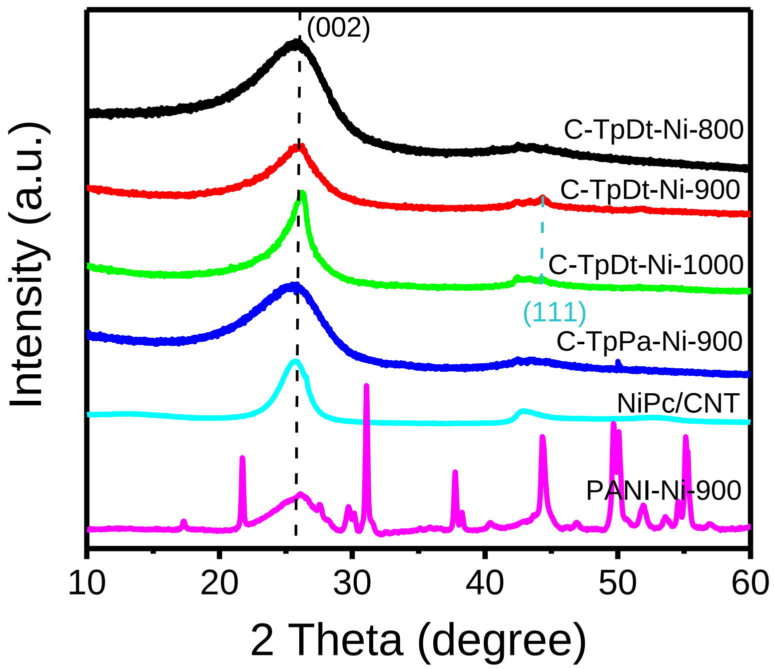


Figure S11. XRD patterns of C-TpDt-Ni-800, C-TpDt-Ni-900, C-TpDt-Ni-1000, C-TpPa-Ni-900, PANI-Ni-900, and NiPc/CNT. The profile of PANI-Ni-900 is identical with our earlier work.[3]

*XPS analysis*

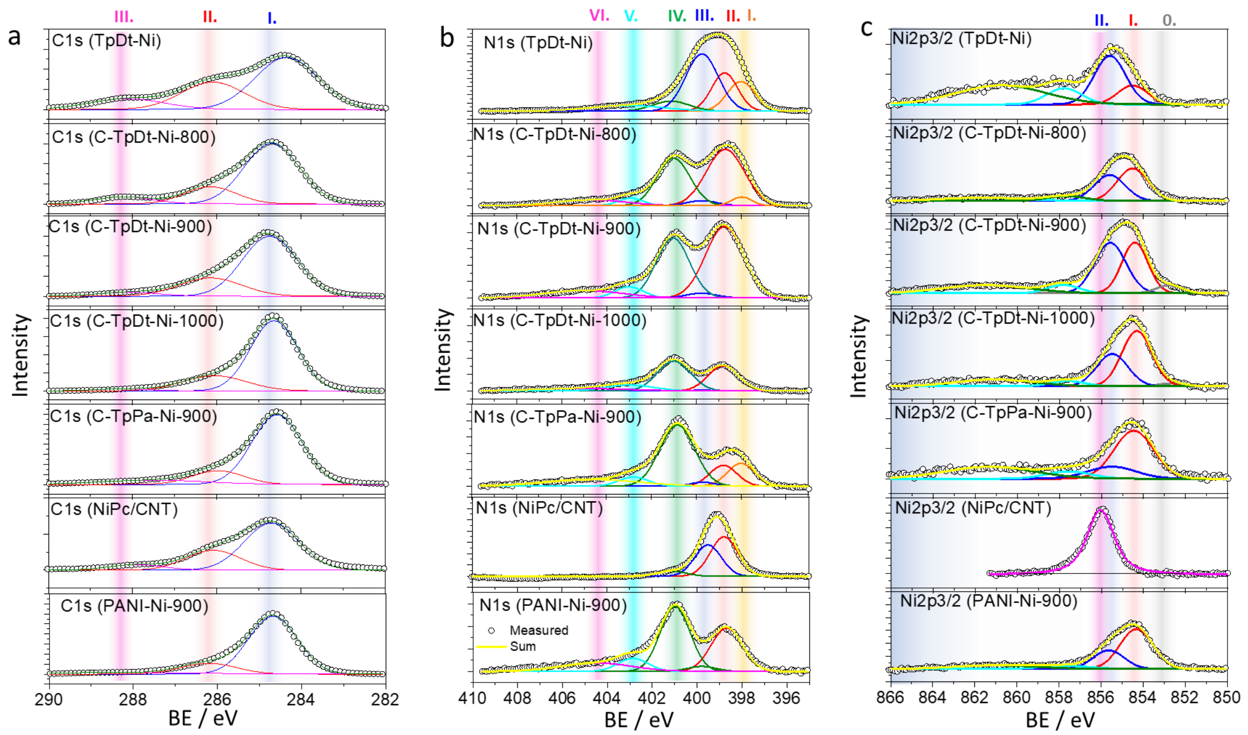


Figure S12. (a) XPS C 1s spectra, (b) XPS N 1s spectra, and (c) XPS Ni 2p spectra for TpDt-Ni, C-TpDt-Ni-800, C-TpDt-Ni-900, C-TpDt-Ni-1000, C-TpPa-Ni-900, NiPc/CNT, and PANI-Ni-900. The PANI-Ni-900 is analogous with our earlier work.[3]

Here, the fitting of the XPS profiles is according to the previous studies.

The C1s spectra are fitted as Ref[11]:

Group I: 284.4 eV to 284.8 eV;

Group II: 286.2 ± 0.2 eV, FWHM ~ 1.5 ± 0.2 eV;

Group III: 288.2 ± 0.2 eV, FWHM ~ 1.5 ± 0.2 eV;

The N1s spectra are fitted following the Ref[12]:

Group I: 398.1 ± 0.2 eV, FWHM ~ 1.5 ± 0.2 eV;

Group II: 398.7 ± 0.2 eV, FWHM ~ 1.5 ± 0.2 eV;

Group III: 399.6 ± 0.2 eV, FWHM ~ 1.5 ± 0.2 eV;

Group IV: 401 ± 0.2 eV, FWHM ~ 1.5 ± 0.2 eV;

Group V: 402.8 ± 0.2 eV, FWHM ~ 1.5 ± 0.2 eV;

Group VI: > 404 eV.

The Ni2p spectra are fitted following the Ref[13]:

Group 0: 852.6 ± 0.2 eV;

Group I: 854.4 ± 0.2 eV, FWHM ~ 1.5 ± 0.2 eV;

Group II: 855.3 ± 0.2 eV, FWHM ~ 1.5 ± 0.2 eV; *

* The chemical state of NiPc/CNT is ~0.5 eV positive than other candidates, consistent with previous studies.[13c, 14]

Note:

In the C1s spectra, it is seen that the intensities for carbon atoms bound to hetero-atoms (C=O/C=N at 288.2 ± 0.2 eV, C-O/C-N at 286.2 ± 0.2 eV, Figure S12a) drop along with increasing pyrolysis temperatures. Aromatic ring formation was revealed by the peak shift from 284.4 eV to 284.8 eV.[11]

The fitted high-resolution N 1s profiles of the pyrolyzed catalyst precursors are shown in Figure S12b (detailed fitted parameters are presented in Table S3-5). The fitted group I (398.1 ± 0.2 eV) and group II (398.7 ± 0.2eV) cover two sp2-hybridized nitrogens bound to carbons, such as imine, pyridinic, or triazinic type N. The group III (399.6 ± 0.2 eV) indicates sp2 hybridized N in Metal-N coordination (Ni-N), OC-NH-C partial double bonds, or multiple graphitic N motifs in a single aromatic ring. Group IV (401 ± 0.2 eV) and V (402.8 ± 0.2 eV) cover the in-plane N-H, graphitic N(-H), and out-of-plane N-H, such as protonated-pyridinic, pyrrolic, graphitic, and quaternary N. Species in group VI (404 eV) should be assigned to oxide N moieties.[12] Clearly, the pyrolysis temperature controls the resulting N species in all C-TpDt-Ni samples. In the unpyrolyzed TpDt-Ni sample, the group I and III signals can be assigned to imine and amine groups. The pyrolysis treatment significantly transformed those into the in-plane sp2 hybridized N moieties.

**Table S3.** Content (at%) of Ni, N, C and O elements for all the samples as measured by XPS.

| **Samples** | Ni | N | C | O |
|---|---|---|---|---|
| TpDt-Ni | 0.50 | 15.15 | 66.24 | 18.11 |
| TpPa-Ni | 0.35 | 10.09 | 71.81 | 17.74 |
| C-TpDt-Ni-800 | 0.66 | 10.28 | 80.62 | 8.45 |
| C-TpDt-Ni-900 | 0.97 | 11.27 | 82.11 | 5.65 |
| C-TpDt-Ni-1000 | 0.48 | 4.63 | 90.85 | 4.04 |
| C-TpPa-Ni-900 | 0.21 | 5.13 | 89.27 | 5.39 |
| PANI-Ni-900 | 0.47 | 4.8 | 91.22 | 3.51 |
| NiPc/CNT | 0.36 | 3.65 | 95.44 | 0.55 |

**Table S4.** The atomic ratio of various N species fitted by XPS analysis.

| **Samples** | Group I. BE~398eV | Group II. BE~398.7eV | Group III. BE~399.6eV | Group IV. BE~401eV | Group V. BE~403eV | Group VI. BE>=404 |
|---|---|---|---|---|---|---|
| TpDt-Ni | 18.32 | 22.14 | 41.75 | 8.23 | 9.56 | 0 |
| TpDt-Ni-800 | 3.81 | 46.12 | 3.27 | 33.82 | 4.08 | 8.89 |
| TpDt-Ni-900 | 0 | 47.08 | 2.56 | 34.49 | 5.52 | 10.39 |
| TpDt-Ni-1000 | 0 | 33.06 | 1.06 | 42.50 | 12.59 | 10.79 |
| TpPa-Ni-900 | 14.43 | 13.11 | 2.24 | 49.18 | 6.25 | 14.79 |
| PANI-Ni-900 | 0 | 30.88 | 3.12 | 43.55 | 8.89 | 13.50 |
| NiPc/CNT | 0 | 52.34 | 40.53 | 7.12 | 0 | 0 |

**Table S5.** The atomic ratio of various Ni species fitted by XPS analysis.

| **Samples** | Group 0. BE~852.6eV | Group I. BE~854.4eV | Group II. BE~855.3eV | Satellites BE > 856eV |
|---|---|---|---|---|
| C-TpDt-Ni-800 | 0 | 44.35 | 35.20 | 20.44 |
| C-TpDt-Ni-900 | 5.10 | 32.32 | 38.66 | 23.93 |
| C-TpDt-Ni-1000 | 2.29 | 47.83 | 29.09 | 20.79 |
| C-TpPa-Ni-900 | 0 | 49.19 | 15.04 | 35.77 |
| PANI-Ni-900 | 0 | 55.58 | 24.38 | 20.04 |
| NiPc/CNT | 0 | 0 | 100 (BE~856.1eV) | 0 |

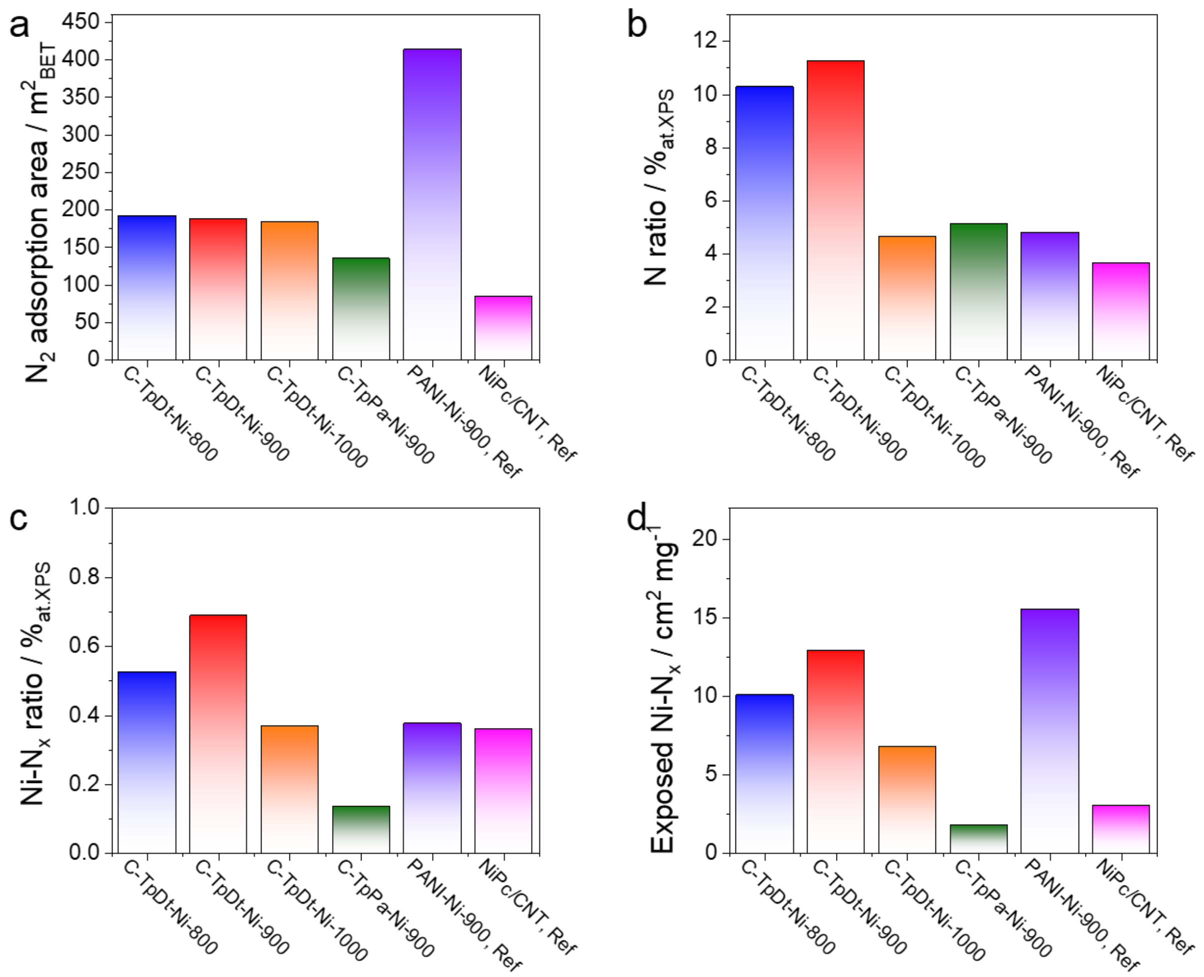


Figure S13. (a) BET derived specific $N_2$ adsorption area, (b) XPS derived surface nitrogen atomic ratio, (c) surface Ni-$N_x$ atomic ratio, estimated upon XPS derived $Ni^{2+}$ and $Ni^{+}$ species ($x_{Ni-Nx}\%_{at.} = x_{Ni^{+}}\%_{at.} + x_{Ni^{2+}}\%_{at.}$), and (d) the surface area contributed by Ni-$N_x$ moieties ($A_{Ni-Nx} = x_{Ni-Nx}\%_{at.} \times\ A_{BET}$; unit is transferred into $cm^2\ mg^{-1}$).

*XAS analysis*

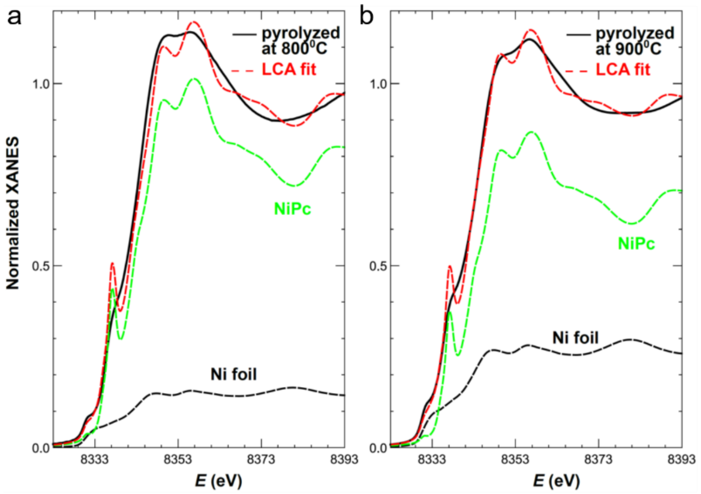


Figure S14. Results of linear combination fitting of experimental Ni K-edge XANES for sample (a) pyrolyzed at 800°C (C-TpDt-Ni-800) and (b) 900°C (C-TpDt-Ni-900). Reference spectra for Ni foil and Ni phthalocyanine used for linear combination analysis are also shown (scaled by their corresponding weight in linear combination).

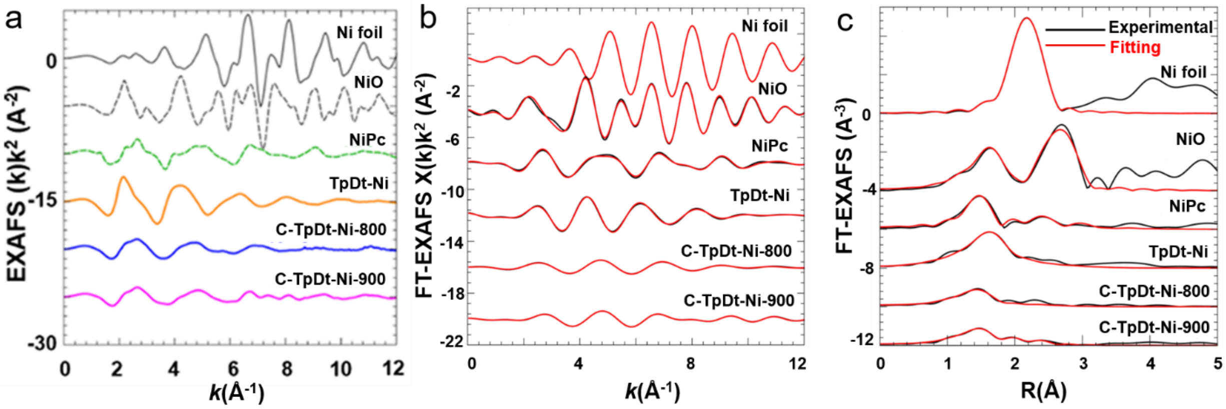


Figure S15. (a) EXAFS spectra for Ni foil, NiO, NiPc, TpDt-Ni, C-TpDt-Ni-800 and C-TpDt-Ni-900. XAFS data fitting in (b) k-space and (c) R-space.

For quantitative EXAFS analysis we perform non-linear least square fitting to theoretical standards, as implemented in FEFFIT code, see Figure S14.[15] Theoretical phases and amplitudes were obtained in self-consistent ab-initio calculations with FEFF8.5 code[16] for reference materials. The complex exchange-correlation Hedin-Lundqvist potential and default values of muffin-tin radii as provided within the FEFF8.5 code were employed.

Fitting of EXAFS spectra $\chi(k)k^2$ was carried out in $R$-space in the range from $R_{\text{min}}$ = 1.0 Å up to $R_{\text{max}}$, where $R_{\text{max}}$ was set to 3.0 Å for NiO and NiPc and to 2.5 Å for metallic Ni, unpyrolyzed Ni COF and Ni COF pyrolyzed at 900$^0$C. For Ni COF pyrolyzed at 800$^0$C we set $R_{max}$ to 1.8 Å to exclude from fitting the contributions beyond the first coordination shells which are too weak in this sample to be reliably identified. In all cases Fourier transform was carried out in the $k$ range from 3.0 Å$^{-1}$ up to 12 Å$^{-1}$.

Guided by the insight from WT-EXAFS analysis in Figure 2c, following paths were included in the fitting: Ni-Ni path for metallic Ni, Ni-O and Ni-Ni paths for NiO, Ni-N, Ni-C and longer Ni-N path for NiPc, Ni-O path for unpyrolyzed sample, Ni-N path for sample pyrolyzed at 800$^0$C, and Ni-N and Ni-Ni paths for sample pyrolyzed at 900$^0$C. For each path the refined parameter were coordination number $N$, bond-length $R$ and disorder factor $\sigma^2$. In addition, correction to photoelectron reference energy $\Delta E_0$ was also fitted. Amplitude reduction factors due to many-electronic processes ($S_0^2$ factors) were estimated based on the fitting of EXAFS samples for reference materials with known coordination numbers.

The results of such fitting are summarized in Figure S15 and Table S6. Good agreement between experimental and modeled data (Figure S15), and low values of fit R-factors (Table S6) give us confidence in the chosen fitting models.

It is challenging to distinguish between EXAFS contributions from elements that are neighbors in Periodic Table (such as N, O and C). Valuable information in this case can be obtained from interatomic distances. As evident from Table S6, significant difference between the bond-lengths in the first coordination shell can be observed for unpyrolyzed and pyrolyzed samples. In the former case, the obtained bond length (2.04 ±0.03 Å) is in a good agreement with Ni-O bond length in NiO reference sample (2.08 ±0.01 Å). In the latter case, the bond between Ni and its nearest neighbor is shorter, (1.87 ±0.01 Å), and agrees with Ni-N bond length in NiPc (1.879 ±0.009 Å). Thus one can conclude that in the pyrolyzed samples (unlike the unpyrolized one), the main Ni-containing structural motifs are Ni-$N_x$ units, similar to those in NiPc. Moreover, for unpyrolyzed sample the 1$^{st}$ shell coordination number is close to 6, suggesting octahedral coordination of Ni species in this sample, in agreement with conclusions from XANES analysis. For

pyrolyzed samples, the 1st shell coordination number is significantly lower, making plausible hypothesis that Ni-$N_4$ structural motifs are dominating moieties.

In addition to these structural motifs, the existence of metallic Ni clusters in pyrolyzed samples is confirmed by EXAFS data fitting. In particular, for sample pyrolyzed at 900 $^0$C the inclusion of Ni-Ni scattering path was found to be necessary to obtain a good fit. The obtained value of Ni-Ni interatomic distance (2.458 ±0.006 Å) is close to Ni-Ni distance in fcc metallic nickel (2.481 ±0.001 Å). The fact that Ni-Ni distance is slightly shorter in pyrolyzed Ni COF sample in comparison to that in bulk Ni metal, may imply the small sizes of formed Ni clusters and/or their strongly disordered nature.

Due to the low contribution of Ni-Ni scattering path, the obtained Ni-Ni coordination number has large uncertainty and cannot be used for a reliable estimation of the concentration of metallic Ni clusters. However, the fraction of metallic Ni can be estimated indirectly from Ni-N coordination number. Since the measured EXAFS signal is averaged over all Ni species in the sample, in the case when Ni-$N_x$ motifs coexist with metallic Ni, the Ni-N coordination number obtained in the EXAFS fitting $N_{Ni-N}$ differs from the true number of N neighbors $\widetilde{N}_{Ni-N}$, and is related to the concentration of metallic Ni $w$ as $N_{Ni-N} = (1 - w)\widetilde{N}_{Ni-N}$. Assuming that in Ni-$N_x$ motifs Ni is coordinated with 4 N atoms (i.e., $\widetilde{N}_{Ni-N} = 4$), the fraction of metallic Ni can be estimated as $1 - N_{Ni-N}/4$. As a result, we can estimate that in the sample pyrolyzed at 800$^0$C, concentration of metallic Ni is ca. 22%, while in the sample pyrolyzed at 900$^0$C concentration of metallic Ni increased to ca. 33%. These estimates are in a good agreement with the aforementioned estimates from XANES analysis. Good agreement between EXAFS and XANES results indicates the validity of our assumption that Ni-$N_4$ motifs are the main N-$N_x$ species in the pyrolyzed catalysts.

**Table S6.** Structure parameters (coordination numbers N, interatomic distances R, disorder factors $\sigma^2$), obtained in fitting of experimental Ni K-edge EXAFS data. Uncertainties of the last digit are given in parentheses.

| Sample | $N_{Ni-C/N/O}$ | $R_{Ni-C/N/O}$ (Å) | $\sigma^2_{Ni-C/N/O}$ (Å$^2$) | $N_{Ni-Ni}$ | $R_{Ni-Ni}$ (Å) | $\sigma^2_{Ni-Ni}$ (Å$^2$) | $\Delta E_0$ (eV) | *R* factor |
|---|---|---|---|---|---|---|---|---|
| Ni foil | - | - | - | 12 | 2.481(1) | 0.0064(1) | 2.8(1) | 0.1% |
| NiO | 6 | 2.08(1) | 0.009(2) | 12(2) | 2.95(1) | 0.007(2) | 3.4(8) | 1.3% |
| NiPc (1st shell, Ni-N) | 4 | 1.879(9) | 0.001(1) | - | - | - | 2.1(9) | 2.0% |
| NiPc (2nd shell, Ni-C) | 8 | 2.95(2) | 0.003(3) | | | | | |
| NiPc (3rd shell, Ni-N) | 6 | 3.32(4) | 0.007(7) | | | | | |
| TpDt-Ni | 6.9(7) | 2.04(3) | 0.010(2) | - | - | - | 1(2) | 0.6% |
| C-TpDt-Ni-800 | 3.1(4) | 1.87(1) | 0.008(2) | - | - | - | -3(2) | 0.8% |
| C-TpDt-Ni-900 | 2.8(2) | 1.875(6) | 0.0079(9) | 1(1) | 2.458(6) | 0.0017(9) | -0.7(8) | 0.2% |

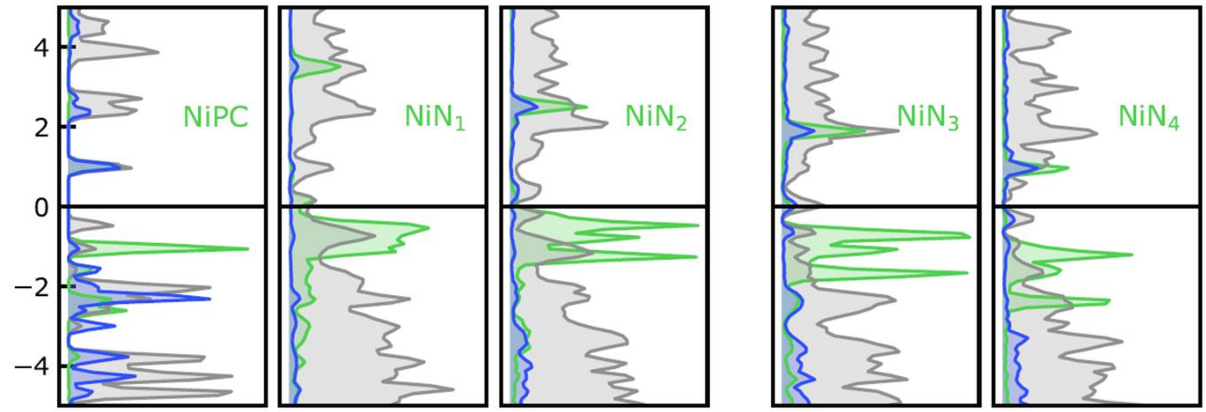


Figure S16. Projected density of state (PDOS) of our studied Ni-N-C catalysts. Those are NiPc (Phthalocyanine), DV-Ni-N1, DV-Ni-N2, DV-Ni-N3, and DV-Ni-N4.

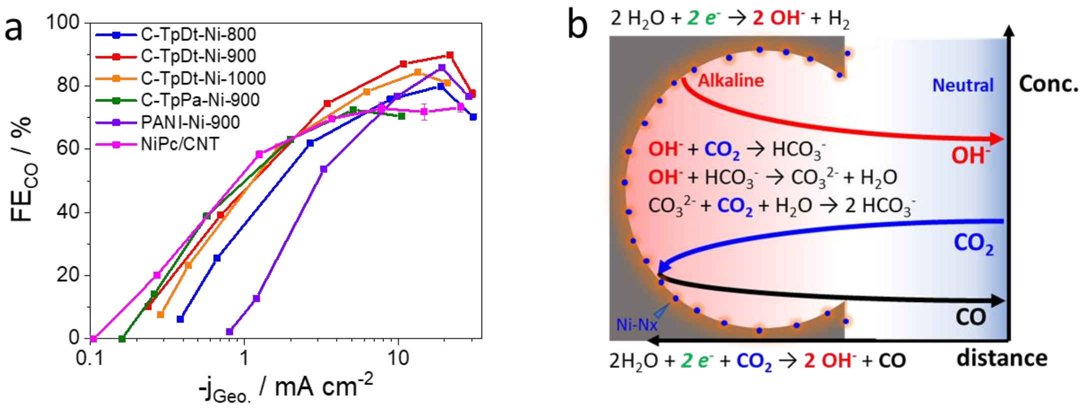


Figure S17. (a) $FE_{CO}$ as a function of overall geometric current density on various Ni-N-C catalysts. (b) Schematic illustration of $CO_2$ mass transfer limitation due to in-pores $OH^-$ formation (by electrochemical $H_2$ and CO evolution) and retention.

# Reference


[1] C. Li, J. Yang, P. Pachfule, S. Li, M.-Y. Ye, J. Schmidt, A. Thomas, *Nature Communications* **2020**, *11*, 4712.

[2] X. Zhang, Y. Wang, M. Gu, M. Y. Wang, Z. S. Zhang, W. Y. Pan, Z. Jiang, H. Z. Zheng, M. Lucero, H. L. Wang, G. E. Sterbinsky, Q. Ma, Y. G. Wang, Z. X. Feng, J. Li, H. J. Dai, Y. Y. Liang, *Nature Energy* **2020**, *5*, 684-692.

[3] T. Möller, W. Ju, A. Bagger, X. Wang, F. Luo, T. Ngo Thanh, A. S. Varela, J. Rossmeisl, P. Strasser, *Energy & Environmental Science* **2019**.

[4] G. Kresse, J. Furthmüller, *Physical Review B* **1996**, *54*, 11169-11186.

[5] G. Kresse, D. Joubert, *Physical Review B* **1999**, *59*, 1758-1775.

[6] B. Hammer, L. B. Hansen, J. K. Nørskov, *Physical Review B* **1999**, *59*, 7413-7421.

[7] A. Hjorth Larsen, J. Jørgen Mortensen, J. Blomqvist, I. E. Castelli, R. Christensen, M. Dułak, J. Friis, M. N. Groves, B. Hammer, C. Hargus, E. D. Hermes, P. C. Jennings, P. Bjerre Jensen, J. Kermode, J. R. Kitchin, E. Leonhard Kolsbjerg, J. Kubal, K. Kaasbjerg, S. Lysgaard, J. Bergmann Maronsson, T. Maxson, T. Olsen, L. Pastewka, A. Peterson, C. Rostgaard, J. Schiøtz, O. Schütt, M. Strange, K. S. Thygesen, T. Vegge, L. Vilhelmsen, M. Walter, Z. Zeng, K. W. Jacobsen, *Journal of Physics: Condensed Matter* **2017**, *29*, 273002.

[8] H. J. Monkhorst, J. D. Pack, *Physical Review B* **1976**, *13*, 5188-5192.

[9] J. K. Nørskov, J. Rossmeisl, A. Logadottir, L. Lindqvist, J. R. Kitchin, T. Bligaard, H. Jónsson, *The Journal of Physical Chemistry B* **2004**, *108*, 17886-17892.

[10] A. J. Medford, C. Shi, M. J. Hoffmann, A. C. Lausche, S. R. Fitzgibbon, T. Bligaard, J. K. Nørskov, *Catalysis Letters* **2015**, *145*, 794-807.

[11] K. Artyushkova, *Journal of Vacuum Science & Technology A* **2020**, *38*, 031002.

[12] aK. Artyushkova, B. Kiefer, B. Halevi, A. Knop-Gericke, R. Schlogl, P. Atanassov, *Chem Commun* **2013**, *49*, 2539-2541; bK. A. S. Kabir, B. Kiefer, P. Atanassov, *Phys. Chem. Chem. Phys.* **2015**, *17*, 17785-17789; cK. Artyushkova, A. Serov, S. Rojas-Carbonell, P. Atanassov, *The Journal of Physical Chemistry C* **2015**, *119*, 25917-25928; dF. Luo, A. Roy, L. Silvioli, D. A. Cullen, A. Zitolo, M. T. Sougrati, I. C. Oguz, T. Mineva, D. Teschner, S. Wagner, J. Wen, F. Dionigi, U. I. Kramm, J. Rossmeisl, F. Jaouen, P. Strasser, *Nature Materials* **2020**, *19*, 1215-1223.

[13] aZ. Jakub, J. Hulva, F. Mirabella, F. Kraushofer, M. Meier, R. Bliem, U. Diebold, G. S. Parkinson, *The Journal of Physical Chemistry C* **2019**, *123*, 15038-15045; bS. Liu, H. B. Yang, S.-F. Hung, J. Ding, W. Cai, L. Liu, J. Gao, X. Li, X. Ren, Z. Kuang, Y. Huang, T. Zhang, B. Liu, *Angewandte Chemie International Edition* **2020**, *59*, 798-803; cH. B. Yang, S.-F. Hung, S. Liu, K. Yuan, S. Miao, L. Zhang, X. Huang, H.-Y. Wang, W. Cai, R. Chen, J. Gao, X. Yang, W. Chen, Y. Huang, H. M. Chen, C. M. Li, T. Zhang, B. Liu, *Nature Energy* **2018**, *3*, 140-147; dL. Soriano, I. Preda, A. Gutiérrez, S. Palacín, M. Abbate, A. Vollmer, *Physical Review B* **2007**, *75*, 233417.

[14] C. Yan, H. Li, Y. Ye, H. Wu, F. Cai, R. Si, J. Xiao, S. Miao, S. Xie, F. Yang, Y. Li, G. Wang, X. Bao, *Energy & Environmental Science* **2018**, *11*, 1204-1210.

[15] B. Ravel, M. Newville, *Journal of Synchrotron Radiation* **2005**, *12*, 537-541.

[16] A. L. Ankudinov, S. D. Conradson, J. Mustre de Leon, J. J. Rehr, *Physical Review B* **1998**, *57*, 7518-7525.
